# TRIM2, a novel member of the antiviral family, limits New World arenavirus entry

**DOI:** 10.1371/journal.pbio.3000137

**Published:** 2019-02-06

**Authors:** Nicolas Sarute, Nouhou Ibrahim, Bani Medegan Fagla, Madakasira Lavanya, Christian Cuevas, Spyridon Stavrou, Guliz Otkiran-Clare, Henna Tyynismaa, Jorge Henao-Mejia, Susan R. Ross

**Affiliations:** 1 Department of Microbiology and Immunology, UIC College of Medicine, Chicago, Illinois, United States of America; 2 Department of Microbiology, Perelman School of Medicine, University of Pennsylvania, Philadelphia, Pennsylvania, United States of America; 3 Department of Biological Sciences, UIC, Chicago, Illinois, United States of America; 4 Research Program for Molecular Neurology, University of Helsinki, Helsinki, Finland; 5 Department of Pathology and Laboratory Medicine, Perelman School of Medicine, University of Pennsylvania, Philadelphia, Pennsylvania, United States of America; University of Texas Medical Branch at Galveston, UNITED STATES

## Abstract

Tripartite motif (TRIM) proteins belong to a large family with many roles in host biology, including restricting virus infection. Here, we found that TRIM2, which has been implicated in cases of Charcot–Marie–Tooth disease (CMTD) in humans, acts by blocking hemorrhagic fever New World arenavirus (NWA) entry into cells. We show that *Trim2*-knockout mice, as well as primary fibroblasts from a CMTD patient with mutations in *TRIM2*, are more highly infected by the NWAs Junín and Tacaribe virus than wild-type mice or cells are. Using mice with different *Trim2* gene deletions and TRIM2 mutant constructs, we demonstrate that its antiviral activity is uniquely independent of the RING domain encoding ubiquitin ligase activity. Finally, we show that one member of the TRIM2 interactome, signal regulatory protein α (SIRPA), a known inhibitor of phagocytosis, also restricts NWA infection and conversely that TRIM2 limits phagocytosis of apoptotic cells. In addition to demonstrating a novel antiviral mechanism for TRIM proteins, these studies suggest that the NWA entry and phagocytosis pathways overlap.

## Abstract 2

TRIM2, one of the proteins mutated in Charcot Marie Tooth Disease, blocks the entry of new world arenaviruses into cells, a novel mechanism for antiviral TRIM proteins, which generally restrict viruses at other stages of infection.

## Author summary

New World arenaviruses (NWAs) are rodent-transmitted viruses that cause high mortality when they evolve the ability to infect humans. Although these clade B pathogenic viruses are known to bind to transferrin receptor 1 and other receptors on the cell surface, the steps leading to their entry into the cell are not well determined. We show that a host factor identified in a previous small interfering RNA (siRNA) screen, tripartite motif 2 (TRIM2), limits NWA endocytosis into cells. Moreover, we show that a member of the TRIM2 interactome, signal regulatory protein α (SIRPA), which is well-known for inhibiting phagocytosis by macrophages, interacts with TRIM2 and also blocks NWA infection. This finding suggests that there are common mechanisms that regulate virus endocytosis and phagocytosis.

## Introduction

Arenaviruses are enveloped single-stranded RNA viruses whose entry is mediated by the viral glycoprotein (GP), generated by proteolytic processing of a precursor into the envelope proteins GP1, GP2, and stable signal peptide (SSP), a third subunit required for virus–cell fusion [1]. The clade B New World arenaviruses (NWAs), including Junín and Machupo viruses—the causative agents of Argentine and Bolivian hemorrhagic fever, respectively—use human but not mouse (*Mus*) transferrin receptor 1 (TfR1) for cell entry [2], whereas the Old World arenaviruses (OWAs) Lassa virus and lymphocytic choriomeningitis virus (LCMV) use alpha-dystroglycan [3]. NWAs also enter cells via TfR1-independent means and use receptors other than TfR1 to infect sentinel cells of the immune system, their probable initial in vivo targets [4–6]. Other clade B NWAs, such as Tacaribe virus, use TfR1s from their own host but not the human receptor (reviewed in [7]). The T-cell immunoglobulin and mucin (TIM) receptor has also been implicated in mediating entry via binding of phosphatidyl serine on the virus membrane (reviewed in [8]), and we suggested that voltage-gated calcium channels (VGCCs) serve as additional NWA entry receptors [9]. Subsequent to GP interaction with receptors on the cell surface, trafficking to a late endosomal compartment is required for virus entry [10–13]. Although it is generally accepted that OWAs enter cells via a macropinocytosis-like process that is clathrin- and dynamin-independent, whether this is also the case for NWAs is less clear [7, 14, 15].

In a small interfering RNA (siRNA) screen for host factors that play a role in Junín virus entry, we identified a number of host genes that alter infection, including tripartite motif 2 (TRIM2), which was antiviral [9]. TRIM2 knockdown resulted in a 3- to 5-fold increase in infection levels by the replication-competent vaccine strain of Junín virus (Candid 1) and by gammaretrovirus pseudoviruses bearing either the Junín (Parodi strain) or Machupo GP as the only NWA protein, suggesting that TRIM2-mediated restriction works at an entry step [9]. TRIM2 did not affect entry by pseudoviruses bearing retroviral envelope proteins or the rhabdovirus vesicular stomatitis virus (VSV) GP [9].

The human genome encodes at least 70 TRIM proteins, many of which function as antiviral restriction factors acting at different stages of the virus replication cycle, including uncoating, transcription, and virion release, as well as indirectly by playing a role in cellular antiviral responses [16–19]. TRIM proteins are characterized by an N-terminal RBCC domain, consisting of a RING domain with potential ubiquitin E3 ligase activity, 1–2 zinc-binding B-box motifs, and a central coiled-coil (CC) domain involved in protein–protein interaction. The C-terminal domains of TRIM proteins are more variable, with approximately 10 different motifs present in the various family members. TRIM2 belongs to subgroup VII, which contains filamin (FIL) domains and NCL-1, HT2A, and Lin-41 (NHL) repeats at their C terminus; only 4 mammalian TRIM proteins belong to this subgroup: TRIM2, TRIM3, TRIM32, and TRIM71 [20]. Little is known about the biology of these 4 proteins. TRIM3 has been implicated in the transport of cellular cargo [21], TRIM71 in microRNA and mRNA biology [22, 23], and TRIM32 is thought to play a role in muscle filaments; mutations in TRIM32 are associated with limb-girdle muscular dystrophy [24].

TRIM2 is highly expressed in the brain. As with other TRIMs, the TRIM2 RING domain encodes E3 ubiquitin ligase activity. TRIM2 binds neurofilament light chain (NEFL) subunit through its RBCC and FIL domains [25]. Knockout mice deficient in TRIM2 were reported to develop NEFL buildup in central nervous system axons accompanied by progressive neurodegeneration, tremor, and ataxia, which was attributed to an inability to degrade NEFL [25]. TRIM2 has also been implicated in rare peripheral neuropathies in humans, part of the Charcot–Marie–Tooth diseases (CMTDs); patients lacking functional TRIM2 protein developed peripheral axonal neuropathy [26, 27].

TRIM2 interacts with several other cellular proteins. It interacts with Bcl-interacting mediator of cell death (BIM/BCL2l11) and regulates its degradation in the proteasome and with myosin5A (MYO5A) through its NHL domain [28, 29]. In a yeast 2-hybrid screen, TRIM2 was also shown to bind signal regulatory protein α (SIRPA/SHPS1) [30]. SIRPA is a transmembrane glycoprotein that plays a critical role in the phagocytosis of cells by macrophages; binding of SIRPA on phagocytic cells to CD47 on the surface of target cells inhibits their engulfment [31]. SIRPA’s cytoplasmic domain contains 4 tyrosine motifs that, when phosphorylated, become binding sites for the SH2 domains of SHP-1 and SHP-2, which in turn get activated, initiating a cascade that blocks phagocytosis. Phosphorylation of SIRPA is regulated by various growth factors and integrin activation [32].

Here, using *Trim2*-knockout mice with different deletions, we show that TRIM2 functions in vivo to suppress NWA infection. Moreover, we show that TRIM2 reduces virus uptake into cells and that one of its interacting partners, SIRPA, functions as an antiviral factor. In in vitro and in vivo studies, we found that TRIM2’s antiviral activity at minimum requires the FIL domain and not the RING domain encoding ubiquitin ligase activity. These studies thus define a novel antiviral function for TRIM proteins and suggest a link in mechanism between virus endocytosis and phagocytosis.

## Results

### TRIM2 restricts NWA infection

**Fig 1:**
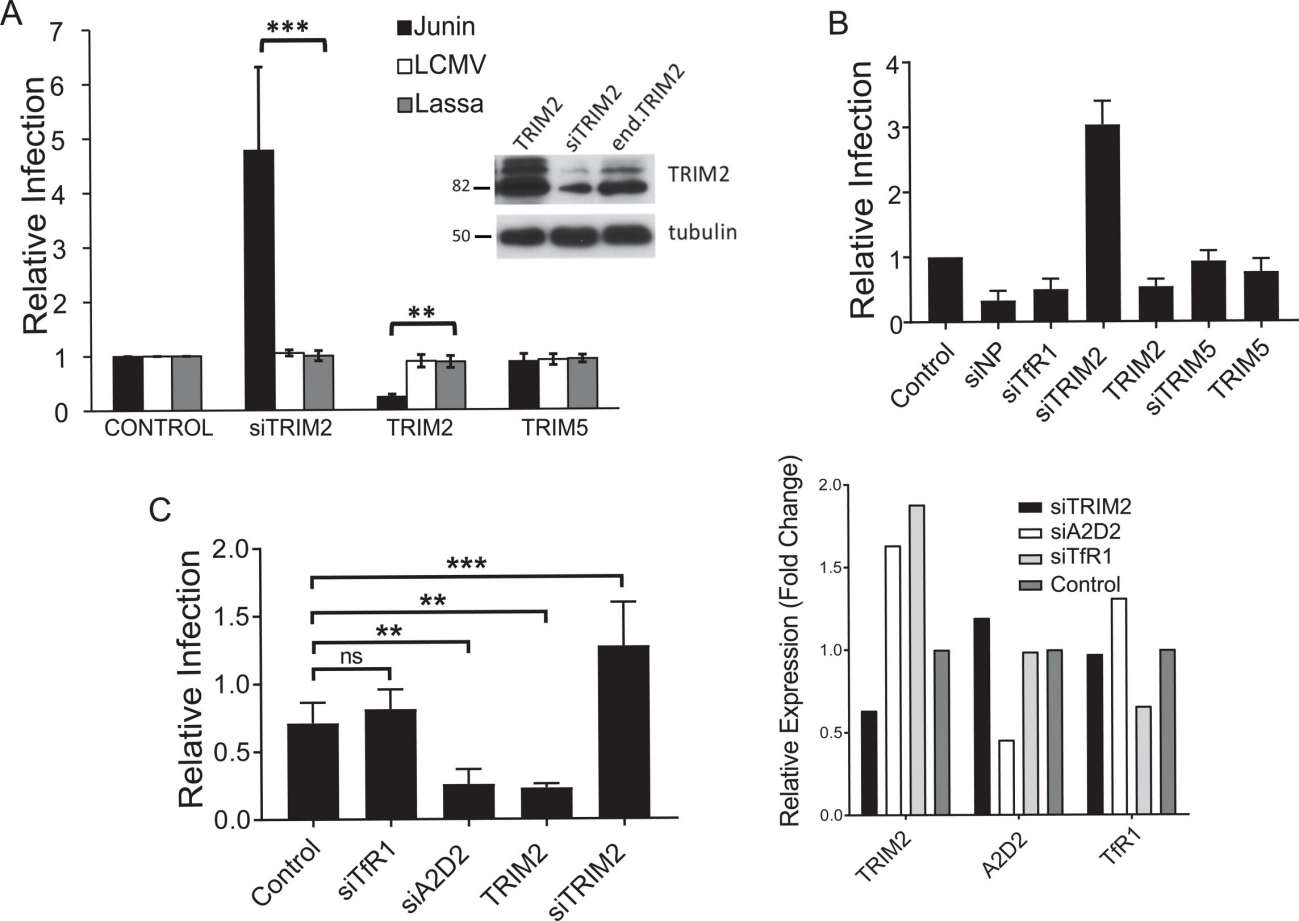
Overexpression of TRIM2 decreases Junín virus but not OWA infection. (A) U2OS cells were transfected with TRIM2 or TRIM5α expression vectors and 24 hr later were infected with Junín virus, Lassa virus, or LCMV GP-pseudoviruses containing the luciferase gene. The data shown are the average and SD of 3 independent experiments. Western blot is from U2OS cells and those transfected with the TRIM2 expression vector or siRNA. Blots were probed with anti-TRIM2 and anti-β-tubulin antisera. (B) U2OS cells were transfected with the indicated siRNAs or TRIM expression vectors and 24 hr later were infected with Candid 1 (MOI 0.1). Reverse-transcribed RT-qPCR for the nucleoprotein RNA was analyzed. Values represent the mean ± SD in 2 independent experiments with triplicate experimental replicates. Control refers to cells treated with a control siRNA. (C) U2OS cells were transfected with the indicated siRNAs and expression vectors and infected with Tacaribe virus; the panel on the right shows the knockdown of each gene. The data shown represent the average and SD of 3 independent experiments. One-way ANOVA was used to determine significance. **P ≤ 0.005; ***P ≤ 0.0005. A2D2, calcium channel subunit α2δ2; LCMV, lymphocytic choriomeningitis virus; MOI, multiplicity of infection; OWA, Old World arenavirus; RT-qPCR, real-time quantitative PCR; siRNA, short interfering RNA; TfR1, transferrin receptor 1.

We previously showed that siRNA-mediated depletion of TRIM2 in human U2OS or 293T cells resulted in increased infection by either Junín or Machupo virus GP-pseudotyped murine leukemia virus (MLV) or the Junín vaccine strain Candid 1 [9]. To determine if TRIM2 overexpression also altered infection, we transfected a TRIM2 expression vector into U2OS cells and then infected them with pseudoviruses bearing the Junín GP (Fig 1A) or with Candid 1 (Fig 1B). Western blot analysis of extracts made from cells transduced in parallel confirmed TRIM2 overexpression and knockdown, respectively (inset, Fig 1A). TRIM2 overexpression resulted in decreased infection by Junín pseudoviruses as well as Candid 1, and as we showed previously, treatment with TRIM2 siRNA increased infection (Fig 1A and 1B). Depletion of either TfR1 (the viral entry receptor in human cells) or the viral nucleoprotein (NP) resulted in decreased Candid 1 infection (Fig 1B). As a control, we tested knockdown and overexpression of the retrovirus restriction factor TRIM5α and showed that it did not alter infection by Junín pseudoviruses or Candid 1 (Fig 1A and 1B) [9].

We also tested whether TRIM2 affected infection by the NWA Tacaribe virus and pseudoviruses bearing the GPs from the OWAs Lassa virus and LCMV. TRIM2 overexpression or knockdown had no effect on Lassa or LCMV GP pseudovirus infection (Fig 1A). In contrast, TRIM2 knockdown increased and overexpression decreased infection by Tacaribe virus (Fig 1C). Knockdown of the calcium channel α2δ2 (CACNA2D2) subunit of the VGCC, which we previously showed was needed for infection by NWAs but not OWAs, reduced infection by Tacaribe virus, whereas TfR1 knockdown had no effect on Tacaribe infection, as this virus does not use this receptor on human cells (Fig 1C) [33, 34]. Thus, TRIM2 preferentially restricts infection by NWAs.

### TRIM2-knockout mice are more susceptible to infection

**Fig 2:**
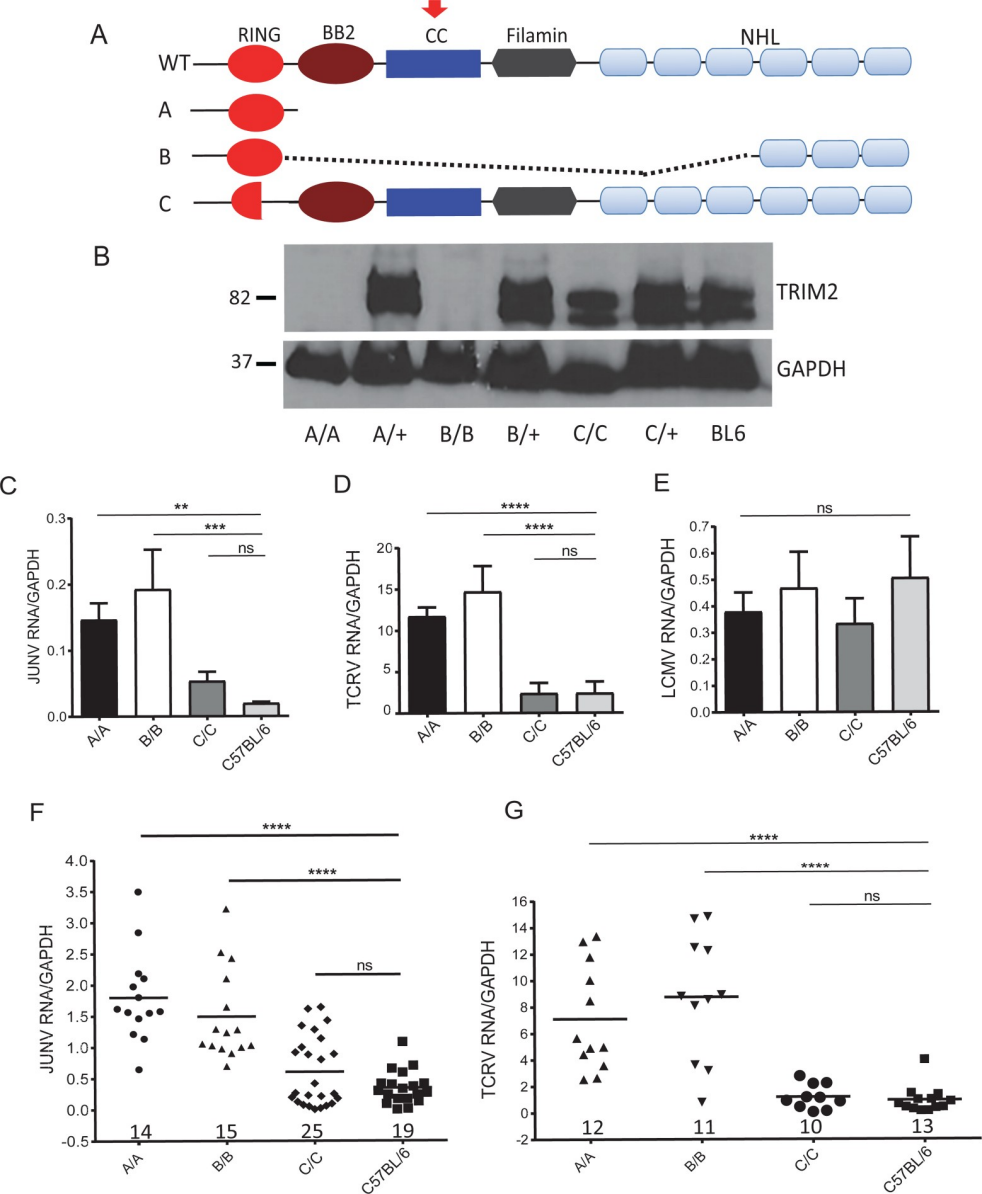
TRIM2 knockout mice are more susceptible to infection with new world arenaviruses. (A) Diagram of the *Trim2* WT and deletion alleles in strains A, B, and C. The red arrow indicates the epitope recognized by anti-TRIM2 antisera. See also S1A and S1B Fig. (B) Western blot analysis of brain extracts from the different homozygous and heterozygous mouse strains, using TRIM2 antisera to an epitope in the CC domain (red arrow in panel A). See also S1C Fig. (C, D, and E) Primary bone marrow–derived macrophages from the different knockout strains were infected with Candid 1, TCRV, and LCMV, respectively, and analyzed by RT-qPCR for viral RNA levels at 24 hpi. Shown are the averages ± SD of 3 different experiments. One-way ANOVA was used to determine significance. **P ≤ 0.005; ***P ≤ 0.0006. See S2A Fig for infection of fibroblasts. (F) Mice of the indicated genotype were infected by intracranial inoculation with 2 × 10^4^ PFU of Candid 1, and at 5 dpi RNA isolated from brains was analyzed for viral RNA. See S2B Fig for virus titers. (G) Mice of the indicated genotype were infected intraperitoneally with 2 × 10^3^ PFU of TCRV by intraperitoneal injection, and at 7 dpi, RNA isolated from spleen was analyzed for viral RNA. *P* values were determined by unpaired *t* tests; ****P ≤ 0.0001. Number of mice in each group is shown above the x-axis. See S2C Fig for virus titers. dpi, days post infection; GAPDH, glyceraldehyde-3-phosphate dehydrogenase; hpi, hours post infection; JUNV, Junín virus; LCMV, lymphocytic choriomeningitis virus; ns, not significant; PFU, plaque-forming units; RT-qPCR, real-time quantitative PCR; TCRV, Tacaribe virus; WT, wild type.

Mice and murine cells can be infected by both the pathogenic and vaccine strains of Junín virus, although mouse TfR1 does not function as a receptor [4, 5, 35, 36]. To determine if TRIM2 acted as an in vivo restriction factor, we created mice with targeted deletion of *Trim2*, using clustered regularly interspaced short palindromic repeat (CRISPR)/CRISPR-associated 9 (Cas9). Two guide RNAS were used, one targeting exon 3 and the other targeting exon 9 (S1A Fig). Three independent strains were developed from the knockout injections: A, which deleted sequences between the 2 guide RNAs and potentially expresses only the RING domain because of a stop codon introduced by the deletion; B, which contains the RING domain but has a large internal deletion and then goes back in frame and retains the 3 terminal NHL repeats; and C, which deleted 30 amino acids, including the C’-terminal portion of the RING domain, and then retains the rest of the protein (Fig 2A). Western blot analysis of brains from these mice, using an antibody that recognizes the CC domain, showed no protein from strains A and B and a slightly smaller protein in strain C (Fig 2B). There were 2 TRIM2 isoforms detected in the wild-type and C extracts, likely the result of alternative splicing of a first coding exon or to protein modification (see below). Although no protein was detected in strains A and B with this antibody, RT-qPCR analysis using primers to exon 11/12 showed that both made RNA containing this region (S1C Fig). We also subcloned the cDNAs for the deleted *Trim2* in strains B and C and showed that they encoded proteins of the predicted sizes (see below). Both the A and B strains developed ataxia and tremors, as had been previously reported for a TRIM2 knockout generated by insertional mutagenesis [25], although the phenotype in A strain mice was more severe. A and B strain mice also developed peripheral neuropathy. Although it lacked part of the RING domain needed for ubiquitin ligase function, strain C had no visible phenotype.

We first tested primary bone marrow–derived macrophages (BMDMs) and fibroblasts from these mice for their ability to be infected by Candid 1, Tacaribe virus, and LCMV. BMDMs from both the A and B knockout strains were infected at about 10-fold higher levels with Candid 1 than were those from parental C57BL/6 mice (Fig 2C); fibroblasts derived from the knockout mice were also more highly infected (S2A Fig). Tacaribe virus also infected BMDMs from the A and B knockout mice at about 5-fold higher levels (Fig 2D), whereas infection by LCMV was similar in knockout and wild-type cells (Fig 2E).

We then tested whether in vivo infection would be affected by TRIM2 deletion. We showed previously that Candid 1 predominantly infects astrocytes and microglia after intracranial inoculation [37]. Mice of each genotype received intracranial inoculations of Candid 1, and 5 d post infection (dpi), their brains were harvested and analyzed for viral RNA levels and virus titers. Both the A and B knockouts showed significantly higher levels of infection than did C57BL/6 mice (Figs 2F and S2B). Similar results were obtained when newborn mice received intraperitoneal inoculations of Tacaribe virus, and their spleens were examined for infection (Figs 2G and S2C). Infection of strain C mice in vivo with either Candid 1 or Tacaribe virus was not significantly different than that seen with C57BL/6 mice (Figs 2C, 2D and S2D and S2E).

These data demonstrated that TRIM2 restricted NWA but not OWA infection in mice as well as in human cells and suggested that the RING domain was not critical for the antiviral activity.

### Fibroblasts from a patient with compound TRIM2 mutations are more susceptible to Junín virus infection

**Fig 3:**
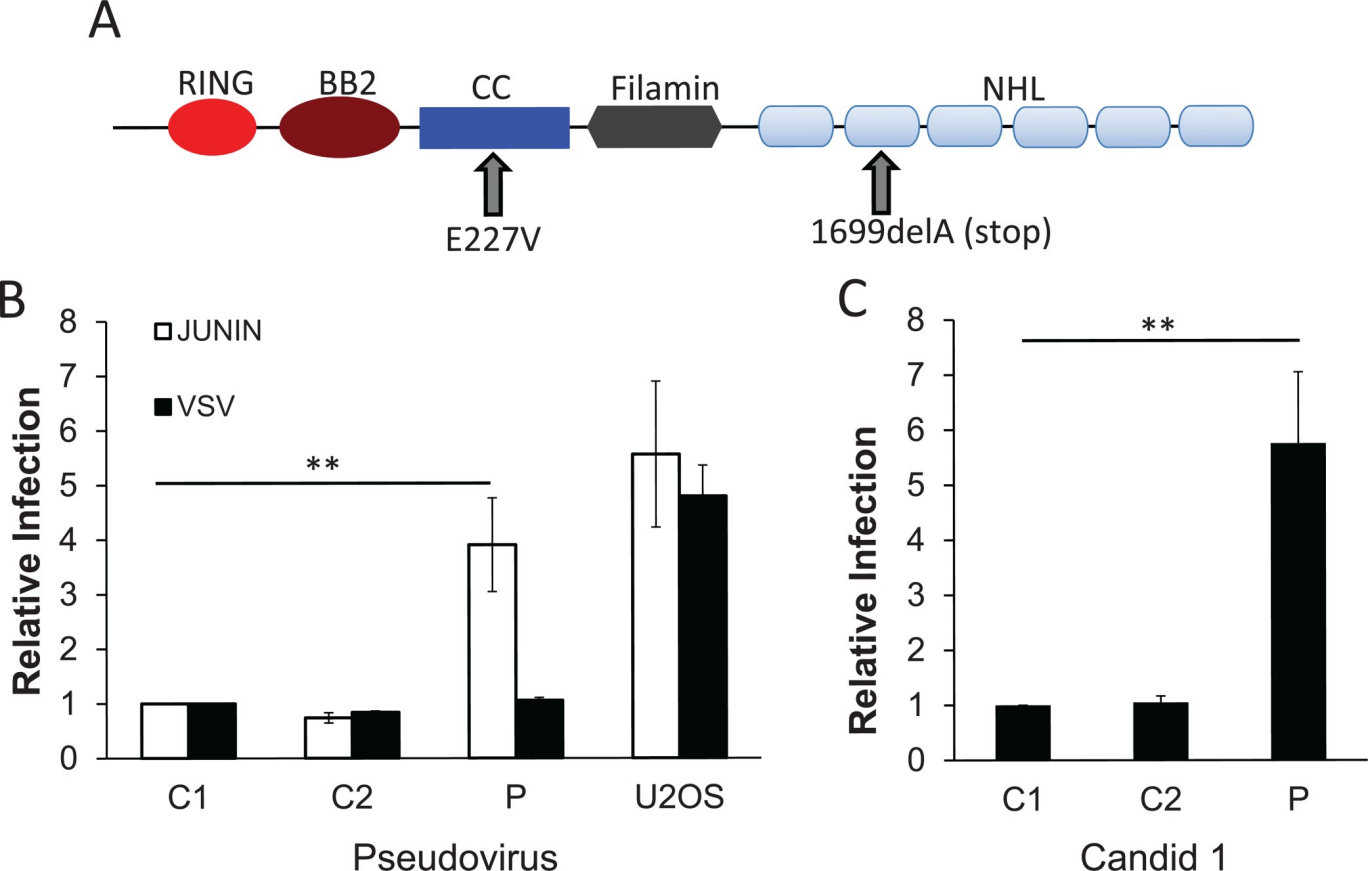
Cells from a Charcot–Marie–Tooth disease patient with TRIM2 mutations are more susceptible to Junín virus infection. (A) Diagram of the mutations found in the TRIM2 alleles (see [26] for more details). (B) Primary fibroblasts from patient (P) or 2 different control patients (C1, C2) were infected with Junín GP or VSV G MLV pseudoviruses encoding the luciferase protein and analyzed for luciferase activity 48 hpi. (C) The same fibroblasts were infected with Candid 1 and analyzed for viral RNA levels by RT-qPCR at 24 hpi. Shown are the averages ± SD of 3 different experiments with passages 4, 5, and 6 of the cells. *P* values were determined by unpaired *t* tests; **P ≤ 0.005. CC, coiled-coil; hpi, hours post infection; MLV, murine leukemia virus; RT-qPCR, real-time quantitative PCR; VSV, vesicular stomatitis virus.

A CMTD patient with early onset peripheral axonal neuropathy was identified as a compound heterozygote for mutations in *TRIM2* by whole-exome sequencing [26]. One allele in this patient contains a missense mutation (E227V) in a conserved stretch of amino acids at the junction of the first CC motif and the intercoil region that destabilizes the protein; the other allele has a 1-bp deletion (c. 1699delA) leading to a frameshift with premature termination, truncating the NHL repeat region and destabilizing the RNA (Fig 3A). RNA from fibroblasts established from this patient showed that TRIM2 protein levels were about 13% of control cells [26]. We tested these primary fibroblasts, as well as those from 2 independent controls, for their ability to be infected with Junín virus. The patient fibroblasts were 4-fold more susceptible to Junín pseudovirus (Fig 3B) and 6-fold more susceptible to Candid 1 (Fig 3C), whereas VSV pseudoviruses showed similar infection levels for all cells. Thus, both human and mouse cells lacking TRIM2 are more susceptible to Junín virus infection.

### TRIM2 decreases virus internalization

**Fig 4:**
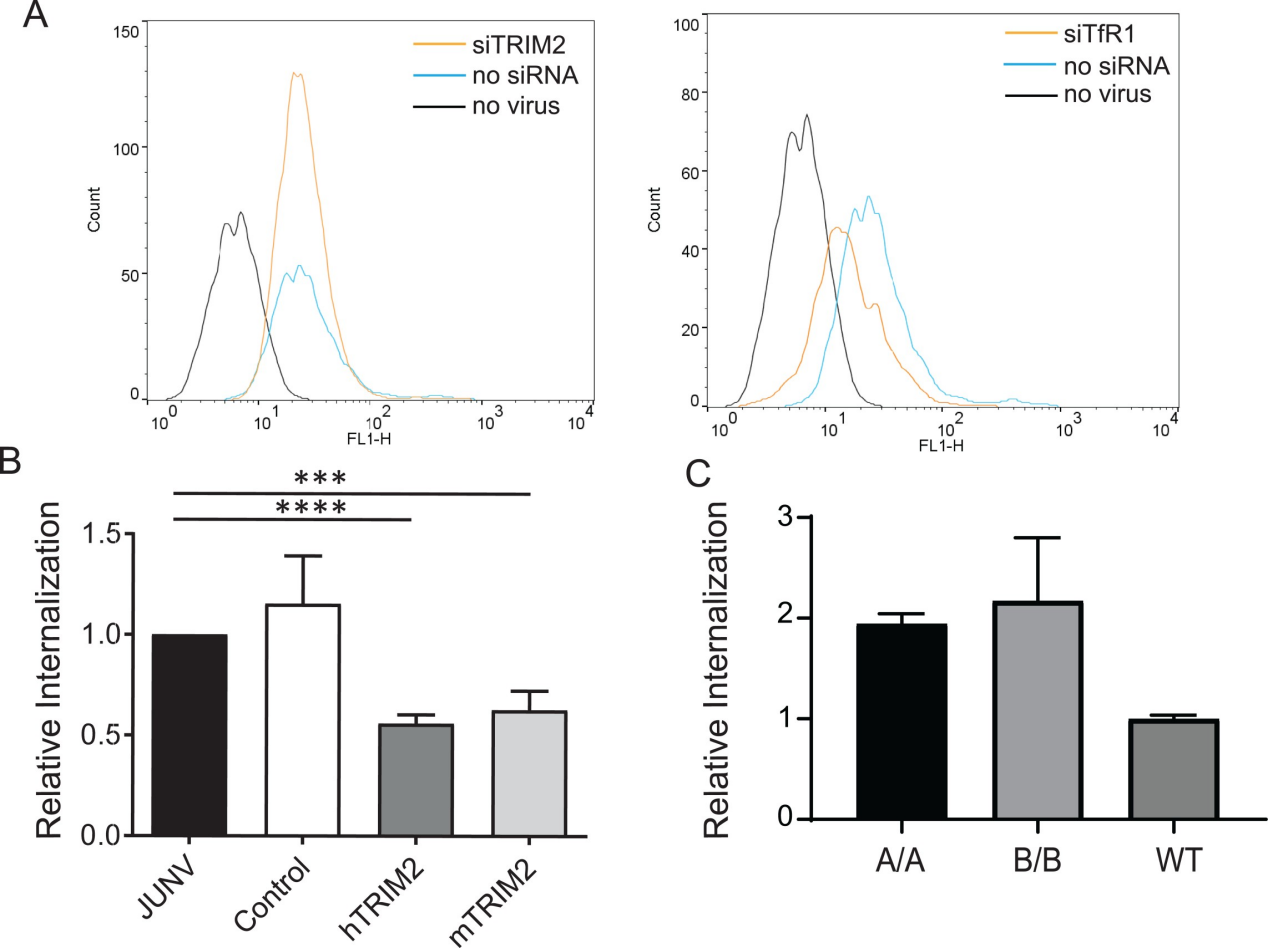
TRIM2 decreases JUNV entry into cells. (A) TRIM2 knockdown does not affect virus binding to cells. U2OS cells were transfected with a TRIM2 siRNA and incubated with FITC-labeled Candid 1. Shown is a representative FACS plot. This experiment was performed twice with similar results. (B) Cells overexpressing hTRIM2 or mTRIM2 or GFP (Control) were incubated with Candid 1, and after a 1-hr incubation at 37°C, virus was stripped from cells, and RNA was isolated and analyzed for viral RNA by RT-qPCR. Shown are the averages ± SD of 6 independent experiments. *P* values were determined by unpaired *t* tests; ***P ≤ 0.0002; ****P ≤ 0.0001. See also S4 Fig. (C) The same experiment was performed with primary bone marrow–derived macrophages isolated from mice of the indicated genotype. Values represent the mean ± SD in 2 independent experiments with triplicate experimental replicates. FACS, fluorescence-activated cell sorting; FITC, fluorescein isothiocyanate; GFP, green fluorescent protein; hTRIM2, human TRIM2; JUNV, Junín virus; mTRIM2, mouse TRIM2; RT-qPCR, real-time quantitative PCR; siRNA, small interfering RNA; TfR1, transferrin receptor 1; WT, wild type.

Junín virus infection requires binding of the viral GP to the cell surface receptor TfR1 in human cells and to the VGCC in mouse cells. We showed previously that siRNA knockdown of TRIM2 did not alter TfR1 expression or TfR1-mediated uptake of transferrin [9], suggesting that TRIM2 does not alter the normal biological function of TfR1. Although TRIM2 is a cytoplasmic protein, it could have an indirect effect on TfR1 or other surface receptors like the VGCC such that they no longer bind Junín virus. We next performed a virus-binding assay with fluorescein isothiocyanate (FITC)-labeled Candid 1 and U2OS human cells, which express high levels of TfR1, and showed that TRIM2 depletion had no effect on binding (Fig 4A). In contrast, knockdown of TfR1 decreased virus binding to cells, as previously been shown [2, 9] (Fig 4A). Surface expression of the VGCC, likely the NWA receptor in mouse cells, was also unchanged in cells derived from TRIM2-knockout mice (S3A Fig).

These data suggested that TRIM2 inhibited infection at a postbinding step. Junín virus enters cells after endocytosis of receptor-bound virus and requires trafficking to a low-pH compartment where virus–membrane fusion occurs and the capsid enters the cytoplasm [4, 12, 38]. To determine if TRIM2 altered virus internalization, cells were transfected with the mouse or human TRIM2 expression plasmids, and 24 hr post transfection, virus was bound to cells on ice and then allowed to internalize at 37°C for 1 hr or kept on ice. Virus was stripped from cells, and internalized viral RNA levels were determined by RT-qPCR. Viral RNA levels were reduced by 50% in cells overexpressing TRIM2 compared to untransfected cells or cells transfected with a control green fluorescent protein (GFP) expression plasmid (Fig 4B). No virus was detected in the cells kept on ice for the duration of the incubation (S4 Fig). When the same experiment was performed with primary fibroblasts isolated from the A and B strain knockout mice, increased virus entry was seen in the knockout cells compared to the wild-type cells (Fig 4C). These data show that TRIM2 plays a role in restricting virus internalization.

### The TRIM2 FIL domain is important for its antiviral activity

**Fig 5:**
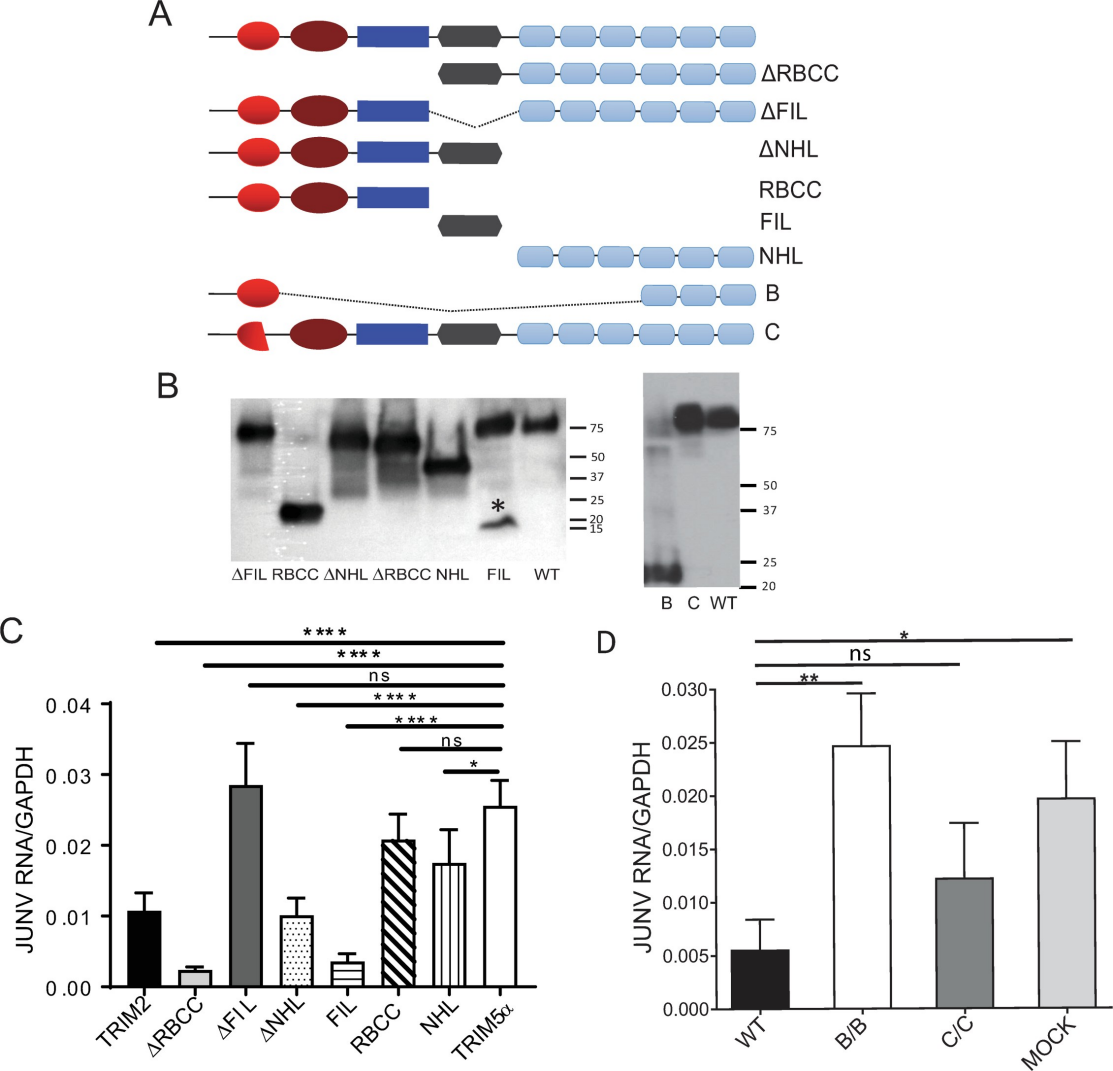
The TRIM2 FIL domain is required for antiviral activity. (A) Diagram of the different deletion constructs. The ΔRBCC, ΔNHL, and NHL constructs were previously described [25]. All constructs were c-myc-tagged. (B) Western blot showing expression of the different deletion constructs. * denotes the monomeric FIL domain. (C) TRIM2 and deletion constructs were transfected into U2OS cells for 24 hr and then infected with Candid 1 (MOI 1). RNA was isolated at 24 hpi and analyzed by RT-qPCR for viral RNA levels. Shown are the averages ± SD of 3 independent experiments. One-way ANOVA was used to determine significance. *P ≤ 0.05; ***P ≤ 0.001; ****P ≤ 0.0001. (D) The same experiment was done with the B and C expression constructs. The average ± SD of 3 independent experiments are shown. One-way ANOVA was used to determine significance. **P ≤ 0.005. GAPDH, glyceraldehyde-3-phosphate dehydrogenase; hpi, hours post infection; JUNV, Junín virus; MOI, multiplicity of infection; ns, not significant; RT-qPCR, real-time quantitative PCR; WT, wild type.

The results presented thus far showed that both mouse and human TRIM2, which are 93% identical at the amino acid level, inhibited NWA infection. Balastik and colleagues created a number of TRIM2 deletion mutants in the mouse backbone and demonstrated that the mouse TRIM2 FIL and NHL domains were both required for NEFL binding (Fig 5A) [25]. We used these and created several additional constructs: one expressing the FIL domain, one expressing the RBCC domain, one deleted for the FIL domain, and constructs encoding the cDNAs from strains B and C (Fig 5A). We also subcloned the strain A protein-coding region but did not detect any stable protein. We then tested these constructs for their antiviral activity. The proteins were all expressed at equivalent levels after transfection into U2OS cells, although the FIL construct appeared to form aggregates (Fig 5B). Transfection of the ΔNHL and ΔRBCC constructs significantly decreased Candid 1 infection, as did the construct retaining only the FIL domain (Fig 5C). In contrast, the ΔFIL construct completely lost antiviral activity (Fig 5C). The construct that expressed only the NHL domain had diminished antiviral activity. We then tested if overexpression of the constructs derived from the strain B and strain C mice would inhibit NWA infection. As we saw with the BMDMs from the mutant mice, the B construct had no antiviral activity against Candid 1, whereas both full-length mouse and human TRIM2 and the C constructs suppressed infection (Fig 5D).

We also tested whether the C construct, which retains antiviral activity but is deleted for part of the RING domain, retained auto-ubiquitinylation activity as was previously reported for TRIM2 [25]. The 293T cells were cotransfected with myc-tagged wild-type TRIM2 or C expression vectors, along with a hemagglutinin (HA)-tagged ubiquitin construct. Following immunoprecipitation with anti-HA, western blots were performed using anti-myc antibodies. The wild-type construct was heavily ubiquitinylated, whereas the C construct showed much lower levels of ubiquitinylation (S1D Fig). Moreover, treatment of primary macrophages from wild-type or strain A mice with the proteasome inhibitor MG132 had no effect on Candid 1 infection of BMDMs (S1E Fig).

Taken together, these data show that the TRIM2’s FIL domain but not its RING domain is necessary for antiviral restriction. Moreover, they confirm that the ubiquitin ligase activity encoded in the RING domain is not needed to inhibit NWA infection.

### TRIM2-interacting protein SIRPA also decreases infection

**Fig 6:**
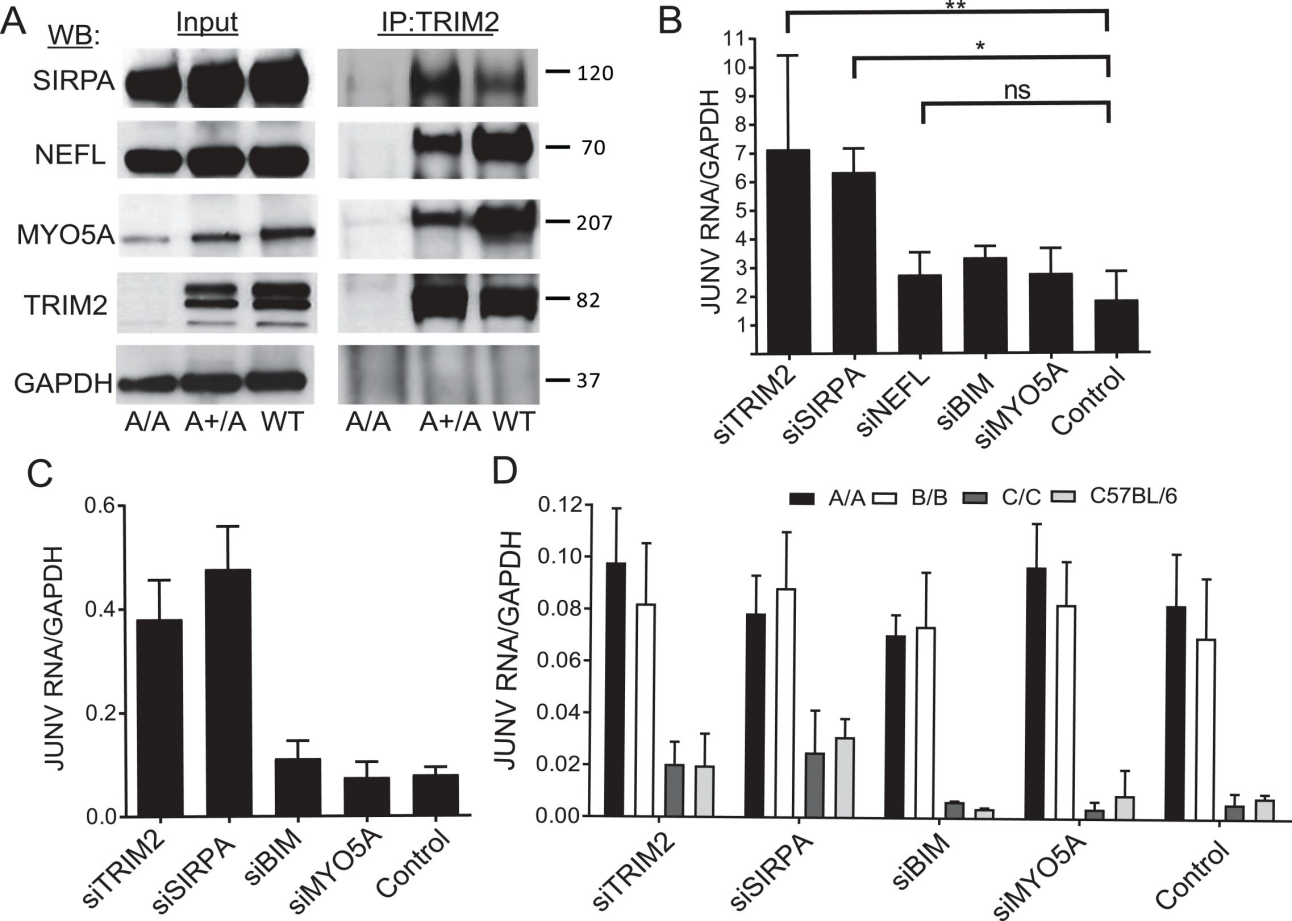
The TRIM2-interacting protein SIRPA also restricts New World arenaviruses. (A) Co-IP of TRIM2-interacting proteins. Brain extracts from strain A knockout, heterozygous, and C57BL/6 (WT) mice were immunoprecipitated with anti-TRIM2 antibody, and WBs were subjected to probing with anti-SIRPA, anti-NEFL, and anti-MYO5A antibodies; anti-GAPDH antibodies served as a control. (B) U2OS and (C) THP-1 cells were transfected with the indicated siRNAs and infected with Candid 1, and RNA was isolated 24 hpi and analyzed for viral RNA. Values in B represent the average of 3–4 independent experiment ± SD. Statistical significance was calculated by one-way ANOVA. *P ≤ 0.01; **P ≤ 0.002. Values in C represent the mean ± SD in 2 independent experiments with triplicate experimental replicates. Knockdowns of the genes in U2OS and THP-1 cells are shown in S5A and S5B Fig, respectively. (D) Primary bone marrow–derived macrophages from mice of the indicated genotype were transfected with the indicated siRNAs. Values represent the average ± SD in 2–3 independent experiments with triplicate experimental replicates. Knockdown of the genes is shown in 5SC Fig. BIM, Bcl-interacting mediator of cell death; GAPDH, glyceraldehyde-3-phosphate dehydrogenase; hpi, hours post infection; IP, immunoprecipitation; JUNV, Junín virus; MYO5A, myosin5A; NEFL, neurofilament light chain; ns, not significant; siRNA, small interfering RNA; SIRPA, signal regulatory protein α; WB, western blot; WT, wild-type.

Interactome studies identified several proteins in addition to NEFL that interact with TRIM2, including SIRPA, BIM, and MYO5A [28–30, 39]. We immunoprecipitated endogenous TRIM2 from the brains of wild-type mice and showed that SIRPA, NEFL, and MYO5A coimmunoprecipitated (Fig 6A). As a control, we showed that none of these proteins precipitated when the anti-TRIM2 antibody was used with strain A brain extracts (Fig 6A). We were unable to carry out these coimmunoprecipitations with BIM and TRIM2 because of high background with the anti-BIM antisera and brain extracts.

To determine if any of these factors also affected NWA infection, we used siRNAs to diminish their expression in U2OS cells. Depletion of SIRPA but not the other proteins caused increased Candid 1 infection (Fig 6B). We also tested whether knockdown of any of these genes would affect infection in human monocyte-like cells, the likely initial targets of Junín virus infection in vivo. THP-1 cells were differentiated with 25 nM of phorbol 12-myristate 13-acetate (PMA) and treated with siRNAs to SIRPA, BIM, and MYO5A. Again, only TRIM2 and SIRPA depletion resulted in increased infection (Fig 6C). SIRPA and TRIM2 knockdown but not BIM or MYO5A also increased Candid 1 infection of primary BMDMs isolated from wild-type or strain C mice (Fig 6D); NEFL expression in THP-1 cells and primary macrophages was undetectable by RT-qPCR, and therefore, siRNA knockdown was not tested. SIRPA overexpression in U2OS cells blocked Candid 1 infection to a similar extent, as TRIM2 overexpression (S6A Fig) and SIRPA knockdown also increased infection by Parodi-GP pseudotyped MLV (S6B Fig). However, knockdown of SIRPA in strain A or B mice did not further increase infection (Fig 6D), nor was SIRPA surface expression diminished in the TRIM2 knockout mice (S3B Fig).

**Fig 7:**
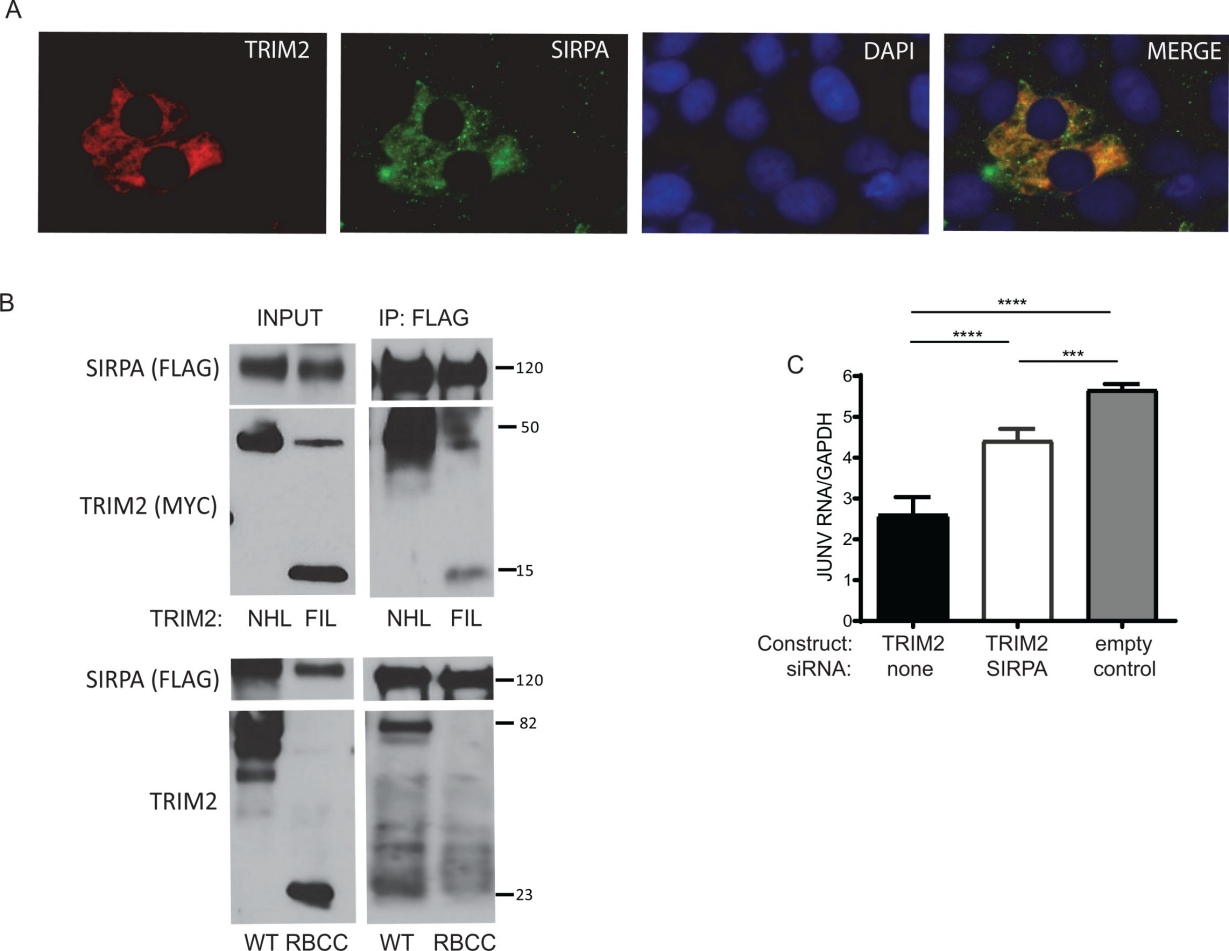
TRIM2 and SIRPA interaction blocks infection. (A) U2OS cells were cotransfected with TRIM2 and SIRPA expression constructs, and immunofluorescence analysis was performed with antibodies against each protein. (B) U2OS cells were cotransfected with myc-tagged TRIM2 or the FIL, NHL, or RBCC constructs (Fig 5A) and FLAG-tagged SIRPA and were immunoprecipitated with anti-FLAG antisera, and blots were subjected to probing with anti-FLAG or anti-myc antisera (top panel) or anti-TRIM2 antisera (bottom panel). (C) U2OS cells were treated with SIRPA siRNA and 24 hr later transfected with the TRIM2 expression vector. Then, 24 hr later, the cells were infected with Candid 1 (MOI 0.1) and reverse-transcribed RT-qPCR for the NP was analyzed. Knockdowns are shown in S6D Fig. Values represent the average of 4 independent experiment ± SD. Statistical significance was calculated by one-way ANOVA. ***P ≤ 0.001; ****P ≤ 0.0001. IP, immunoprecipitation; JUNV, Junín virus; MOI, multiplicity of infection; RT-qPCR, real-time quantitative PCR; WB, western blot; WT, wild type.

SIRPA and TRIM2 also colocalized in transfected U2OS cells (Fig 7A); this colocalization was not affected by NWA infection (S6C Fig). Using the deletion constructs described in Fig 5A, we also found that TRIM2 coimmunoprecipitated via the FIL or NHL but not the RBCC domain (Fig 7B). Finally, to confirm that the TRIM2’s inhibition of infection relied on its interaction with SIRPA, we treated U2OS cells overexpressing TRIM2 with SIRPA siRNA and infected them with Candid 1. SIRPA knockdown in the context of TRIM2 overexpression restored infection levels almost to that seen in control cells (Fig 7C). Taken together, these data suggested that TRIM2 and SIRPA function in the same pathway to restrict NWA internalization.

### SIRPA phosphorylation decreases upon NWA infection

**Fig 8:**
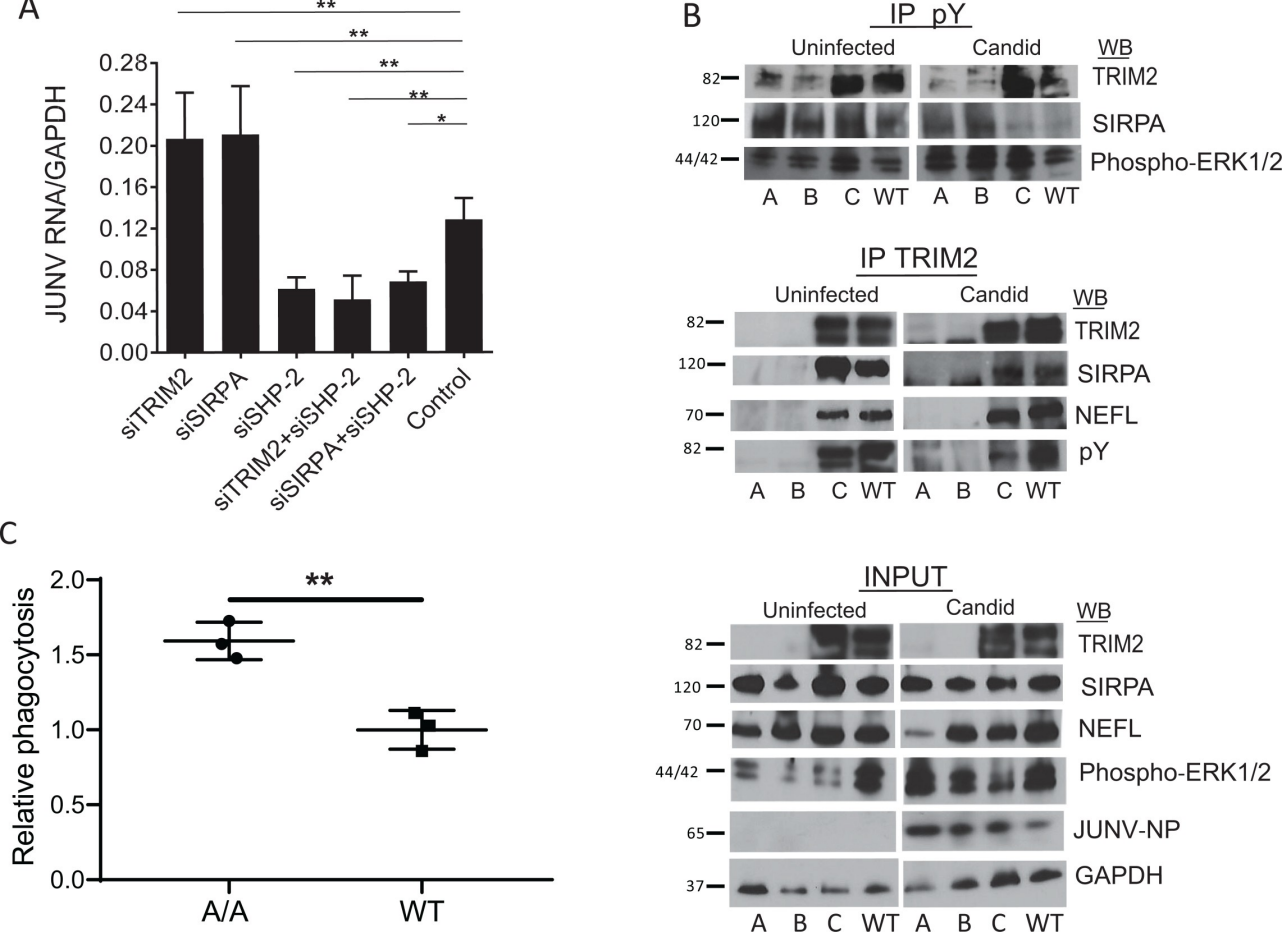
Role for SHP-2 in infection and TRIM2 in inhibition of phagocytosis. (A) U2OS cells were transfected with the indicated siRNAs and infected with Candid 1. Knockdowns of the RNAs are shown in S7B Fig. (B) Extracts from the brains of uninfected and Candid 1–infected mice were prepared and immunoprecipitated with anti-phosphotyrosine or anti-TRIM2 antisera and analyzed by WB with the indicated antibodies. (C) BMDMs isolated from 3 mice of each genotype were incubated with apoptotic phrodo Red–labeled thymocytes. Shown is the average percent internalization in CD11b+ cells for 3 experiments, normalized to WT in each experiment. Statistical significance was determined by unpaired *t* test. **P ≤ 0.005. Representative FACS plots are in S8 Fig. No difference was seen when BMDMs from either genotype were incubated with live thymocytes (S8 Fig). BMDM, bone marrow–derived macrophage; FACS, fluorescence-activated cell sorting; GAPDH, glyceraldehyde-3-phosphate dehydrogenase; IP, immunoprecipitation; JUNV, Junín virus; NEFL, neurofilament light chain; NP, nucleoprotein; Phospho-ERK1/2, phosphorylated extracellular regulated kinase 1/2; siRNA, small interfering RNA; SIRPA, signal regulatory protein α; WB, western blot; WT, wild type.

SIRPA is expressed on the surface of antigen-presenting cells such as macrophages and plays a critical role in phagocytic engulfment of tumor and other cells [31]. Upon binding to CD47 on tumor cells, the cytoplasmic tail of SIRPA becomes tyrosine-phosphorylated, and SHP-1 and SHP-2 phosphatases are recruited and activated, initiating dephosphorylation of downstream substrates [40]. SHP-1 is predominantly expressed in hematopoietic cells, whereas SHP-2 is more ubiquitously expressed. We next tested whether SHP-2 also played a role in regulating NWA infection using siRNA knockdown in U2OS cells. SHP-2 depletion resulted in a large decrease in Candid 1 and Tacaribe virus infection (Figs 8A and S7A).

These data suggested that phosphorylation of SIRPA or TRIM2 might play a role in infection; whereas the biological significance of SIRPA phosphorylation is well-established, TRIM2 phosphorylation has not been previously reported. We thus tested whether endogenous TRIM2 and SIRPA were tyrosine-phosphorylated. Brain extracts from A, B, C, and wild-type mice were immunoprecipitated with anti-phosphotyrosine antisera, and anti-TRIM2 and anti-SIRPA antisera were used to detect protein on western blots. A single TRIM2 isoform, corresponding to the upper band of the doublet, was immunoprecipitated from the extracts from the C and wild-type mice but not the A or B mice (top panel, Fig 8B). SIRPA was also phosphorylated in the brains of all the mice. Similar results were seen when TRIM2 or SIRPA was overexpressed in U2OS cells (S7B Fig). Next, we tested whether infection with Candid 1 altered phosphorylation of TRIM2 or SIRPA. SIRPA phosphorylation was detected in the infected brains of strains A and B but was greatly decreased in strain C or wild-type mice upon infection (Fig 8B). A similar decrease in phosphorylation of endogenous SIRPA was seen after Tacaribe virus infection of TRIM2-transfected U2OS cells (S7D Fig). TRIM2 phosphorylation was not altered by Candid 1 or Tacaribe virus infection. Moreover, when we coimmunoprecipitated TRIM2 and SIRPA, we found that the interaction between TRIM2 and SIRPA decreased upon infection (Figs 8B and S7C). These data suggest that dephosphorylation of SIRPA leads to its decreased interaction with TRIM2.

Finally, we tested whether loss of TRIM2 affected phagocytosis of apoptotic cells by macrophages, a process known to be down-regulated by SIRPA [41, 42]. BMDMs isolated from strain A TRIM2-knockout and wild-type mice were incubated with phrodo Red–labeled apoptotic thymocytes, and relative phagocytosis was analyzed; phrodo Red–labeled viable thymocytes served as a control. BMDMs from the knockout mice phagocytosed significantly more apoptotic cells than did those from wild-type mice (Figs 8C and S8), suggesting that TRIM2/SIRPA complexes might be fundamental to regulation of different endocytic processes.

## Discussion

Arenavirus infection requires binding of the viral GP to cell surface receptors followed by trafficking to acidic endosomes, where virus fusion occurs and capsids are released into the cytoplasm [38]. Although the general steps in the NWA entry pathway have been elucidated, the cellular proteins involved in this process have not been identified, particularly with regard to factors that might limit virus entry. Here, we show that TRIM2, a member of a relatively understudied TRIM subfamily, acts to limit internalization of NWAs but not OWAs and that it does this by interacting with SIRPA, a protein known to be involved in phagocytosis, a specialized form of endocytosis.

TRIM proteins are known to affect different stages of viral infection, including uncoating, viral gene transcription, release from the cells, and intrinsic/innate immune responses, and many of these activities require the ubiquitin ligase activity conferred by the RING domain [18, 19]. TRIM2 itself has been implicated in the ubiquitination and degradation of several interacting partners through its RBCC domain, including BIM and NEFL [25, 29]. In contrast, we found that TRIM2 inhibition of NWA infection both in vitro and in vivo was independent of the RBCC domain and instead required the FIL domain. Indeed, the C mutant, which lacked auto-ubiquitination through partial deletion of its RING domain, still behaved as a restriction factor in vitro and in vivo. Mice bearing this gene deletion had no neurological disease, suggesting that NEFL degradation also does not play a role in the neuropathology seen in CMTD patients.

Many TRIMs are found in the cytoplasm and do not colocalize with commonly used cellular markers for subcellular compartments such as the Golgi apparatus, endocytic vesicles, clathrin-coated pits, mitochondria, intermediate filaments, tubulin, and actin; the exceptions are TRIM1/midline 2 (MID2) and TRIM18/MID1, which localize to microtubules [43, 44]. TRIM2 belongs to the subgroup of cytoplasmic filamentous TRIMs that also do not colocalize with known compartment markers, including tubulin [44]. The filamentous structures might be involved in cargo transport of virus particles and contribute to TRIM2 restriction activity. For example, TRIM3, another subgroup VII member, plays a role in the cytoskeletal-associated-recycling/transport complex and binds to the kinesin motor protein kinesin family member 21B (KIF21B) as well as MYO5, a microtubule-associated motor protein [21, 45]. TRIM2 also associates with MYO5A (Fig 6A)[28]. Although siRNA knockdown of MYO5A did not affect Junín virus infection in vitro, it is possible that other motor proteins are involved in TRIM2 activity.

**Fig 9:**
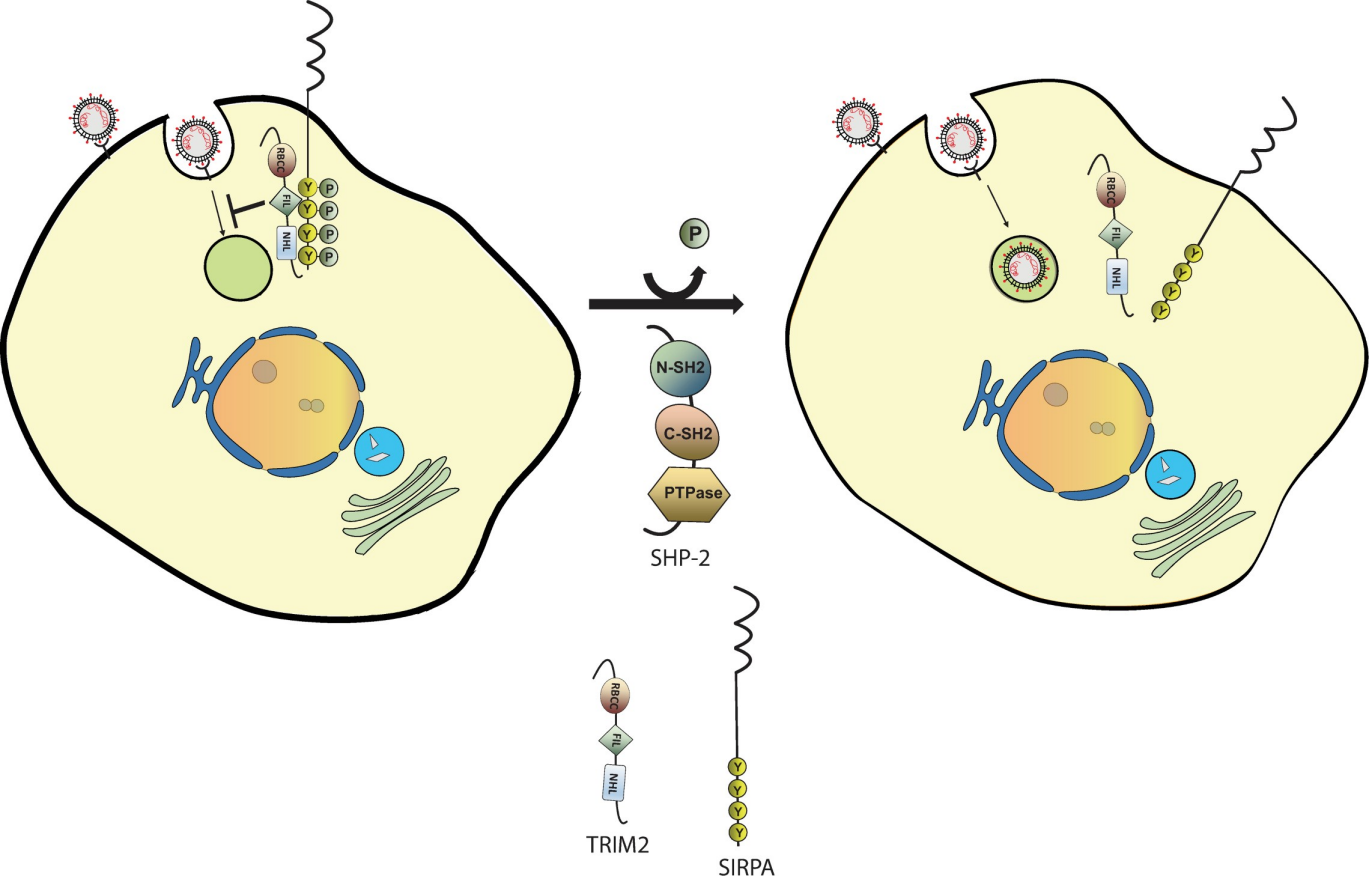
Model for TRIM2–SIRPA inhibition of New World arenavirus infection. TRIM2 and phosphorylated SIRPA form a complex that limits virus endocytosis. Dephosphorylation of SIRPA, possibly by SHP-2, leads to dissociation of the complex and downstream signaling, thereby allowing virus entry to proceed, similar to what is thought to occur when SIRPA-mediated inhibition of phagocytosis is relieved. PTPase, protein phosphatase; SIRPA, signal regulatory protein α.

Of the proteins in the TRIM2-interactome, only SIRPA showed anti-NWA activity. Like TRIM2, SIRPA is expressed in both myeloid and neuronal cells. A major role for SIRPA is the inhibition of phagocytosis upon binding to CD47 on host cells [31, 46]. The cytoplasmic domain of SIRPA contains 4 tyrosine motifs that harbor the consensus binding sites for the SH2 domains of SHP-1 and SHP-2 phosphatases, which upon SIRPA binding subsequently dephosphorylate downstream targets, thereby regulating phagocytosis [31]. Phosphorylation of SIRPA is regulated by various growth factors such as epidermal growth factor and integrin activation and is greatly increased in cells overexpressing catalytically inactive SHP-2 [47]. Our data demonstrated that TRIM2 and tyrosine-phosphorylated SIRPA constitutively interact in vivo and that such interaction is diminished upon Junín virus infection. We also showed that SIRPA phosphorylation is decreased upon infection; although TRIM2 also contains phosphotyrosines, infection did not lead to its dephosphorylation. Whether infection leads to SIRPA dephosphorylation and disassociation from TRIM2 or follows the dissociation is currently under investigation. However, similar interactions have been reported for TRIM2’s interaction with BIM; TRIM2 binds to BIM only when it is phosphorylated by p42/p44 mitogen-activated protein (MAP) kinase [29]. TRIM2 binds to membrane acidic phospholipids found on the cytosolic side of membranes, which may bring it into contact with SIRPA [48]. Taken together, these data suggest that phosphorylated SIRPA binds to TRIM2 and that this complex blocks virus internalization; dephosphorylation of SIRPA, either directly by SHP-2 or by other cellular phosphatases activated by infection, leads to dissociation of the complex and allows infection (Fig 9). Although we have not yet demonstrated how infection triggers this response, we as well as others have shown that arenaviruses interact with several Toll-like receptors (TLRs), and SHP phosphatases have been implicated in both TLR- and retinoic acid–inducible gene I (RIG-I)-mediated signaling [4, 37, 49–52]. However, SHP-2 is involved in many pathways, so the inhibition of infection found in cells depleted for SHP-2 may not be directly linked to its interaction with SIRPA. We also found that loss of TRIM2 lead to increased macrophage engulfment of apoptotic cells, a process known to be regulated by SIRPA, suggesting that there is overlap in the pathways used for NWA entry and phagocytosis.

In conclusion, we show that TRIM2, which belongs to a subfamily in which other members play a role in cargo trafficking, interacts with SIRPA, a known modulator of phagocytosis, and that this interaction plays a role in limiting NWA entry, an antiviral function heretofore not described for TRIM proteins. Whether TRIM2 affects the other known functions of SIRPA, including phagocytosis, is currently under investigation. The results of these studies could lead to a better understanding of its role in macrophage and neuronal cell function in addition to its role in virus entry.

## Materials and methods

### Ethics statement

All mice were housed according to the policies of the Institutional Animal Care and Use Committee of the University of Pennsylvania and of the Animal Care Committee of the University of Illinois at Chicago; all studies were performed in accordance with the recommendations in the Guide for the Care and Use of Laboratory Animals of the National Institutes of Health. The experiments performed with mice in this study were approved by the University of Pennsylvania IACUC (protocol #803700) and University of Illinois at Chicago ACC (protocol #15–222).

### Cell lines and viruses

Vero, U2OS, BHK-21, and 293T cells were cultivated in Dulbecco’s modified Eagle Medium (DMEM; Gibco) supplemented with glutamine (2 mM), 10% fetal bovine serum (FBS; Invitrogen), and penicillin (100 U/ml)-streptomycin (100 μg/ml) (Invitrogen). THP-1 cells were grown in RPMI medium (Gibco) supplemented with 10% FBS and antibiotics. Candid 1 (obtained from Robert Tesh), was propagated in Vero cells, whereas LCMV (obtained from John Wherry) and Tacaribe virus (TRVL-11573; BEI Resources) were propagated in BHK-21 cells. Cells monolayers were infected at 70%–80% confluency with a multiplicity of infection (MOI) of 0.01–0.03. Media were removed 24 hr post infection (hpi), and the cells were fed with media supplemented with 2% FBS. Media were harvested at 3, 4, and 5 dpi to collect LCMV and at 7, 8, 9, and 10 dpi for Candid 1 and Tacaribe virus. Virions were partially purified by centrifugation through a 30% sucrose cushion, resuspended in DMEM supplemented with 2% FBS, and stored at −80°C until use. MLV pseudoviruses encoding the luciferase gene and bearing the different viral GPs were created as previously described [4].

### Virus titration

Candid 1 titers were determined by infectious center assays (ICAs). Vero cells were infected with serial dilutions of the virus for 1 hr at 37°C. Virus was removed, and cells were washed with PBS followed by the addition of an overlay composed of 1% agarose and medium supplemented with 2% FBS. Three days after infection, the cells were fixed with 4% paraformaldehyde, permeabilized with blocking buffer (1X PBS, 2% BSA, 0.1% Triton X-100), incubated with a monoclonal antibody against JUNV NP (NP IC06-BA10; BEI Resources), and incubated with Alexa Fluor 488-coupled secondary antibody (Invitrogen). Cells were visualized with a Keyence fluorescence microscope and foci counted using automated software.

Tacaribe virus titers were determined by TCID_50_ [53]. In brief, virus dilutions from 10^−1^ to 10^−8^ were used to infect Vero cell monolayers cultured in 96-well flat-bottom plates (Corning). The plates were incubated for 1 wk at 37°C, and the virus titer was defined as the last dilution showing cytopathic effects in culture in at least half of the wells infected with each dilution (12 replicates per dilution).

LCMV titers were determined by plaque assay [54]. Briefly, Vero cells were seeded on 6-well plates and infected with serial 10-fold dilutions of LCMV. Agarose overlays (1% agarose in 2X medium 199 [Gibco]) were added to each well after removing the inoculum. The plates were incubated for 4 d at 37°C, fixed with 10% formaldehyde, and stained with 0.1% crystal violet solution, after which plaques were counted.

### Knockout mice

To generate *Trim2*-knockout mice, exon 3 and exon 9 were targeted by 2 sgRNAs using CRISPR/Cas9 technology (S1 Fig). The sgRNAs and CRISPR RNAs were microinjected into zygotes from C57BL/6N mice (Charles River) by the University of Pennsylvania Transgenic and Chimeric Mouse Facility. Genotyping was performed using primers 5′-GCTTTTTCTACTACTTGGTGGCC-3′ and 5′-CCCGTGATTTCTGTGTTAGTTCA-3′; these primers only amplified the A and B knockout alleles, as they are about 25 kB apart in the wild-type gene. To further determine small deletions or mismatches at the endogenous target arising from dsDNA break repair via NHEJ, we performed T7 endonuclease 1 (T7EN1) cleavage assay on genomic DNA. PCR amplification of exon 2 (5′-GCTTTTTCTACTACTTGGTGGCC-3′ and 5′-CCCGTGATTTCTGTGTTAGTTCA-3′) and exon 9 (5′-AGCTTCAGGTTGGTTTCTGGA-3′ and 5′-GACATCATGCAAATGTGAGCAGA-3′). The PCR products were then denatured and reannealed; the annealed PCR products were treated with T7EN1, as recommended by the manufacturer (NEB) and analyzed on 2% agarose gels. The exact deletions found in each strain were determined by sequencing genomic DNA (all strains) and cDNA (strains B and C) generated from total cellular RNA (sequences showing the deletion and coding regions are deposited in a Mendeley dataset at http://dx.doi.org/10.17632/d2vwry7j3x.2).

### Generation of primary murine macrophages

Primary BMDMs were isolated from hind limbs of 8- to 10-wk-old mice as previously described [4]. Macrophages were cultured in DMEM supplemented with 10% FBS, penicillin (100 U/ml)-streptomycin (100 μg/ml), and 100 μg/ml of macrophage colony–stimulating factor (M-CSF; Gibco). Cells were harvested 7 d after plating and were seeded in 24-well plates for siRNA knockdown and infection assays.

### Candid 1 infection of mouse and human macrophages

Mouse macrophages were infected with Candid 1 at a MOI of 1, and after adsorption for 1 hr at 37°C, unbound virus was washed off with 0.1 M sodium citrate (pH 3). THP-1 cells were differentiated into macrophages by treatment with 200 μM PMA (Sigma) for 24 hr. Cells were washed with PBS, fresh media were added, and the cells were incubated at 37°C for 72 hr. Cells were infected with Candid 1 as described above for mouse macrophages.

### Infection of primary human fibroblasts

Three different experiments were performed, using cell passages 4, 5, and 6. Patient and control cells, as well as U2OS cells, were infected with MLV pseudoviruses bearing the Junín or VSV GPs for 48 hr, and luciferase readings were taken to evaluate infection levels. For Candid 1 infections, the cells were infected with Candid 1 (MOI = 0.1) for 24 hr, and RNA was isolated. Reverse-transcribed RT-qPCR was performed for the expression of NP, and the fold infection levels were compared between patient and control fibroblasts. The results of the experiments from the 3 passages were averaged.

### In vivo infections

Eight- to 10-wk-old mice were infected by intracranial inoculation of Candid 1. Each mouse was injected with 2 × 10^4^ PFU, and the infection progressed for 5 d, at which time the brains were harvested. Neonatal mice (1–3 d after birth) were infected with Tacaribe virus (TRVL-11573) by intraperitoneal inoculation. Each pup received 2 × 10^3^ TCID_50_ of the virus. Spleen infection was analyzed at 1 wk post infection.

### Quantification of virus isolated from organs

The brains of Candid 1-infected mice were homogenized in 1X PBS. The homogenate was clarified by centrifugation, and the supernatants were collected and stored at −70°C. Viral titers were quantified by ICA. A portion of the brain homogenate was used for RNA isolation by using the TRIzol reagent (Invitrogen) according to the manufacturers’ instructions. The spleens from Tacaribe virus–infected pups were homogenized and clarified as described for adult mice. Virus titers were determined by TCID_50_.

### RNA isolation and RT-qPCR

Total RNA was isolated using the RNeasy kit (Qiagen). The RNA was used as a template for cDNA synthesis using the SuperScript III First-Strand Synthesis System (Invitrogen) and random hexamer primers following the manufacturer’s specifications. RT-qPCRs were performed with specific primer pairs (S1 Table) using a Power SYBR green PCR kit (Applied Biosystems) and the QuantStudio 5 Real-Time PCR System (Applied Biosystems). RNA quantifications were normalized to glyceraldehyde-3-phosphate dehydrogenase (GAPDH). The amplification conditions were 50°C for 2 min, 95°C for 10 min and 40 cycles of 95°C for 15 s, and 60°C for 1 min. The efficiency of amplification was determined for each primer pair by generating a standard curve with 10-fold serial dilutions of a known concentration of DNA. The slope values of the standard curves for the primer pair amplicons ranged from 3.5 to 3.2, indicating 90%–100% efficiency. For each primer pair, a no-template control was included, and each sample was run in triplicate.

### Fluorescence-activated cell sorting (FACS)

BMDMs from mutant (A/A, B/B, C/C) and wild-type mice were stained with mouse anti-dihydropyridine binding complex (A1S) antibody (Millipore) and FITC-labeled anti-mouse CD172a (SIRPA) (Biolegend). Cells stained with the A1S antibody were incubated with Alexa 647–conjugated secondary antibody (Invitrogen). Cells were analyzed in a CyAn ADP High-speed Analyzer (Beckman Coulter) using FlowJO v10 (Tree Star) software. Gating strategies and fcs files are deposited in a Mendeley dataset at http://dx.doi.org/10.17632/d2vwry7j3x.2.

### Binding assay using FITC-labeled Candid 1

Candid 1 was concentrated by centrifugation on 30% sucrose cushions, titered, and labeled with FITC using Fluorotag FITC conjugation kit (Sigma). Cells were transfected with siRNAs and incubated with FITC-labeled Candid 1 for 1 hr on ice and then transferred to 37°C for 1 hr; a particle/cell ratio of 1,000 was used to ensure saturation of all binding sites. Cells were subjected to the above described protocol and analyzed in a FACS Calibur cytometer (Becton Dickinson).

### Virus entry assay

U2OS cells were transfected in triplicate with the human TRIM2, mouse TRIM2, or pGFP plasmids. At 24 hr post transfection, the cells were incubated on ice with Candid 1 (MOI of 5) for 1 hr, shifted to 37°C for 1 hr, and then treated with sodium citrate (pH 3) at 37°C for 15 min to strip off virus still on the cell surface. RNA was isolated and used for RT-qPCR to measure internalized virus. Values were normalized to Candid 1–infected untransfected cells.

### RNA interference

For the depletion of target genes in human and mouse cells, siRNAs from Qiagen were used for TRIM2 (SI04165602), SHP-2 (SI04165602), and control (1022076); from Ambion for CACNA2D2 (21426), NEFL (17405), BIM (262307), MYO5A (118346), and SIRPA (109944); and from Dharmacon for TfR1 (L-003941). Briefly, cells were transfected using the forward transfection method and Lipofectamine RNAi Max (Invitrogen). siRNA depletion was carried out for 48 hr. Cells were infected with Candid 1 or Junín GP-MLV pseudoviruses, and plates were incubated for another 24 hr.

### Generation of TRIM2 and SIRPA constructs

The c-myc-tagged mouse TRIM2, ΔRBCC, ΔNHL, and NHL constructs were obtained from Martin Balastik [25]. The human TRIM2 and TRIM5α constructs were obtained from Walter Mothes. Constructs encoding the *Trim2* sequence from strains B and C were generated by PCR using reverse-transcribed RNA from mouse brain extracts and amplified with the primers 5′-TGGTGGAAGCTTGCAATGGCCAGTGAGGGCGCCAGCA-3′ and 5′- TGGTGGCTCGAGCTGTAAGTACCGGTAGACCTT-3′. The ΔFIL construct was generated by PCR-mediated plasmid DNA deletion from the full-length TRIM2 plasmid, using primers designed to amplify the entire coding sequence except for the region to be deleted: 5′-CAACCTGGGGACCATCCTCATCCGCTCTGCCGACG-3′ and 5′-GACACGTCGGCAGAGCGGATGAGGATGGTCCCCAGG-3′ [55]. The RBCC construct was generated by PCR using the human TRIM2 plasmid as template and the primers 5′-TTGTTGAAGCTTGCAATGCACAGGAGTGGCCGT-3′ and 5′- TTGTTGTCTAGACTGGTCGGCCAGCTCGTT-3′, and the FIL construct was generated using primers 5′-GGGGTACCATGACCACCAACGCCGTTGC-3′ and 5′-CCTCTAGACACTTTCAGCTTAAACGGGC-3′. The full-length coding sequence of human *SIRPA* was amplified by PCR using cDNA reverse transcribed from U2OS cells RNA with primers 5′-TAATGGGGATCCGCAATGGAGCCCGCCGGCCCG-3′ and 5′-TTGTTGTCTAGACTTGTCGTCATCGTCTTTGTAGTCCTTCCTCTGGACCTGGAC-3′; a FLAG-tag was included in the reverse primer. The purified DNA from each construct was cloned into a pcDNA3.1 (+) *myc*-His vector (Thermo-Fisher); the myc and His tags were in frame with the coding regions of the constructs. The final constructs were validated by Sanger sequencing.

### Western blot analysis

Equal amounts of protein extracts (50 μg) were resolved by 10% SDS-PAGE and transferred to polyvinylidene difluoride (PVDF) membranes. Detection of JUNV NP was done using a monoclonal antibody NA05-AG12 (BEI Resources). Myc-tagged TRIM2 proteins were detected with an anti-Myc antibody (Cell Signaling Technologies [CST]), and FLAG-tagged SIRPA was detected with an anti-FLAG M2 antibody (Sigma). Endogenous and transfected TRIM2 was detected by rabbit anti-TRIM2 antibodies (Sigma SAB4200206). NEFL, SIRPA, MYO5A, SHP-2, and phospho-ERK1/2 were detected with rabbit polyclonal antibodies (CST).

### Transfection of TRIM2 and SIRPA constructs for infection assays

Full-length and mutant versions of TRIM2 and full-length SIRPA plasmids (Addgene) were transfected into U2OS cells using Lipofectamine 3000 (ThermoScientific) for 24 hr according to the manufacturers’ instructions. The cells were infected with Candid 1 or Tacaribe virus (MOI = 10) for 1 hr on ice and shifted to 37°C for an additional hour. The staining and visualization of the cells was performed as described above.

### Ubiquitination assay

Myc-tagged TRIM2 and strain C constructs were cotransfected with an expression plasmid encoding HA-tagged ubiquitin (Addgene) in 293T cells. After 24 hr in culture, 20 μM MG-132 (Sigma) was added to the media, and cells were cultured for 8 hr. Cells were lysed with 1X cell lysis buffer (CST) supplemented with 2% of Halt Protease and Phosphatase Inhibitor Cocktail (ThermoScientific) and 50 mM *N*-ethylmaleimide (NEM; Sigma). Lysates were subjected to immunoprecipitation with a rabbit polyclonal anti-HA antisera (CST) and Protein A/G agarose beads (Santa Cruz Biotechnology) and analyzed by western blot, using anti-myc antibodies (CST).

### Immunofluorescence on cultured cell lines

After transfection of the expression plasmids, the cells were fixed with ice-cold methanol, incubated with 125 mM glycine, permeabilized with 1X PBS-0.3% Triton X-100, and then blocked with 1X PBS-1% BSA. Staining with primary antibodies rabbit polyclonal anti-TRIM2 (Sigma SAB4200206), mouse monoclonal anti-human SIRPA (R&D Systems), and rabbit anti-Myc (CST) was carried out according to the manufacturer’s suggestion. After washing with PBS–0.1% Tween-20, the cells were incubated with Alexa Fluor–coupled (anti-mouse 488, anti-rabbit 568, anti-chicken 647) secondary antibodies (Invitrogen). Cells were visualized under a Keyence fluorescence microscope.

### Immunoprecipitation of endogenous proteins from brains

Brain tissue was homogenized in 1X cell lysis buffer (CST) supplemented with 2% of Halt Protease and Phosphatase Inhibitor Cocktail (ThermoScientific). The protein lysate was incubated on ice for 30 min, sonicated 4 times for 30 s, and clarified by centrifugation. The extracts were precleared with Protein A/G PLUS-Agarose beads (Santa Cruz Biotechnology), and the supernatant was incubated with the primary antibody (rabbit polyclonal anti-TRIM2; Sigma SAB4200282) or mouse Phospho-Tyrosine mAb (P-Tyr-100; CST) and 20 μl of Protein A/G PLUS-Agarose beads overnight. The immunocomplexes were analyzed by western blots as described above.

### Phagocytosis assay

Thymocytes from a wild-type mouse were treated with 0.1 μM dexamethasone (Sigma) for 14 hr at 37°C to induce apoptosis and then stained with pHrodo Red, succinimidyl ester (ThermoScientific) for 1 hr. Fully differentiated BMDMs from 3 mice of each genotype were incubated in duplicate with the thymocytes for 2 hr at 37°C at a ratio of 1:5, after which they were stained with FITC-conjugated anti-CD11b (Invitrogen) and analyzed by FACS. The percentage of double-positive cells was determined, and the percent internalization was normalized to wild type for each experiment. Presented is the average of 3 experiments done on different days.

### Statistical analysis and data

Each experiment was done with 3 technical replicates/experiment. Data shown are the average of at least 3 independent experiments, or as indicated in the figure legends. For in vivo experiments, the number of mice used in each experiment is shown in the graphs. Statistical analysis was performed using the GraphPad/PRISM software. Raw data for all figures are deposited in a Mendeley dataset at http://dx.doi.org/10.17632/d2vwry7j3x.2.

## Supporting information

S1 Fig (A) Diagram showing the position of the guide RNAs used to generate the TRIM2 KO mice. Shown are the genomic deletions found in strains A, B, and C. (B) Diagram of the domains of TRIM2 present in the mutant mice. Shown are the primers used to analyze TRIM2 expression. (C) RT-qPCR analysis of RNA isolated from the brains of strains A, B, and C, using the indicated primers. (D) Ubiquitination assay performed with TRIM2 wild-type and strain C constructs. Immunoprecipitation was with anti-HA (Ub tag) and western blot with anti-myc (TRIM2 tag). (E) Primary macrophages isolated from strain A or C57BL/6 mice were treated with MG132 prior to and during infection. *P ≤ 0.03. One-way ANOVA was used to determine significance. HA, hemagglutinin; KO, knockout; RT-qPCR, real-time quantitative PCR; TRIM2, tripartite motif 2.
(PDF)

S2 Fig (A) Candid 1 infection of fibroblasts derived from strain A, B, and C mice. Shown are the averages ± SD of 3 different experiments. ***P ≤ 0.0005; ****P ≤ 0.0001. (B) Candid 1 titers in the brains of infected mice. Each symbol represents an individual mouse. Shown above the axis are the numbers of mice in each group. **P ≤ 0.003; ***P ≤ 0.0007. (C) Tacaribe virus titers in the spleens of infected mice. *P ≤ 0.02. One-way ANOVA was used to determine significance.
(PDF)

S3 Fig Primary macrophages from the indicated mice were stained with antibodies to the α1S subunit of the VGCC (anti-A1S) (A) and SIRPA (CD172a) (B). Shown below the histograms is the median fluorescence of BMDMs derived from 2 independent mice. BMDM, bone marrow–derived macrophage; SIRPA, signal regulatory protein α; VGCC, voltage-gated calcium channel.
(PDF)

S4 Fig TRIM2 decreases Junín virus entry into cells. The same experiment as described in Fig 4B was performed, except that after virus binding on ice for 1 hr, the cells were incubated at 37°C or left on ice; the virus was stripped of all cells prior to RNA isolation. Shown are the averages ± SD of 3 different experiments. **P ≤ 0.004. One-way ANOVA was used to determine significance. TRIM2, tripartite motif 2.
(PDF)

S5 Fig Knockdown controls for Fig 6. Panel A, Fig 6B; Panel B, Fig 6C (RNA, left; protein, right); Panel C, Fig 6D.
(PDF)

S6 Fig (A) U2OS cells were transfected with TRIM2, TRIM5α, or SIRPA expression vectors and 24 hr later infected with Candid 1 (MOI 0.1). RT-qPCR for the Junín NP was analyzed. Shown are the averages ± SDs of 3 independent experiments. One-way ANOVA was used to determine significance. **P ≤ 0.002; ***P ≤ 0.001. (B) U2OS cells were transfected with SIRPA, TfR1, or control siRNAs for 48 hr and infected with the Junín GP (Parodi)-pseudotyped MLV containing the luciferase gene. The data shown are the average and SDs of 8–10 replicates. One-way ANOVA was used to determine significance. ****P ≤ 0.0001; *P ≤ 0.01. (C) Immunostaining of U2OS cells cotransfected with TRIM2 and SIRPA expression vectors. Shown to the right is the quantification of TRIM2-SIRPA colocalization performed with 5 independent fields of each experiment and analyzed using the Coloc2 algorithm (ImageJ). (D) Knockdown control for Fig 7C (RNA, left; protein, right). GP, glycoprotein; MLV, murine leukemia virus; MOI, multiplicity of infection; NP, nucleoprotein; RT-qPCR, real-time quantitative PCR; siRNA, small interfering RNA; SIRPA, signal regulatory protein α; TfR1, transferrin receptor 1; TRIM, tripartite motif.
(PDF)

S7 Fig (A) U2OS cells were transfected with the indicated siRNAs and infected with Tacaribe virus, and RNA was isolated 24 hpi and analyzed for viral RNA. Values represent the average of 3 independent experiment ± SD. Statistical significance was calculated by one-way ANOVA. ****P ≤ 0.0001; *P ≤ 0.02. (B) Knockdown controls for Figs 8 and S7A. (C) U2OS cells were transfected with TRIM2 expression plasmid ± Tacaribe virus infection (MOI = 1). The extracts were immunoprecipitated with anti-phosphotyrosine antisera and analyzed by western blots with anti-myc (TRIM2) and a rabbit polyclonal anti-SIRPA. hpi, hours post infection; MOI, multiplicity of infection; TRIM2, tripartite motif 2.
(PDF)

S8 Fig Representative FACS plot of BMDMs isolated from strain A and wild-type mice incubated with phrodo Red–labeled apoptotic (DEX-treated) and viable thymocytes (live) (see Fig 8C). BMDM, bone marrow–derived macrophage; DEX, dexamethasone; FACS, fluorescence-activated cell sorting.
(PDF)

S1 Table Primer pairs used for reverse-transcribed RT-qPCR. RT-qPCR, real-time quantitative PCR.
(DOCX)

## Abbreviations

BIM/BCL2l11: Bcl-interacting mediator of cell death
BMDM: bone marrow–derived macrophage
CACNA2D2: calcium channel subunit α2δ2
Cas9: CRISPR-associated 9
CC: coiled-coil
CMTD: Charcot–Marie–Tooth disease
CRISPR: clustered regularly interspaced short palindromic repeat
dpi: days post infection
FIL: filamin
FITC: fluorescein isothiocyanate
GFP: green fluorescent protein
GP: glycoprotein
HA: hemagglutinin
KIF21B: kinesin family member 21B
LCMV: lymphocytic choriomeningitis virus
MAP: mitogen-activated protein
MID: midline
MLV: murine leukemia virus
MYO5A: myosin5A
NEFL: neurofilament light chain
NHL: NCL-1, HT2A, and Lin-41
NP: nucleoprotein
NWA: New World arenavirus
OWA: Old World arenavirus
PMA: phorbol 12-myristate 13-acetate
PTPase: protein phosphatase
RIG-I: retinoic acid–inducible gene I
siRNA: small interfering RNA
SIRPA: signal regulatory protein α
SSP: stable signal peptide
TfR1: transferrin receptor 1
TIM: T-cell immunoglobulin and mucin
TLR: Toll-like receptor
TRIM: tripartite motif
VGCC: voltage-gated calcium channel
VSV: vesicular stomatitis virus
