# Supplementary figures and images for: TRIM2, a novel member of the antiviral family, limits New World arenavirus entry
Source: PLoS Biol. 2019 Feb 6;17(2):e3000137. doi: 10.1371/journal.pbio.3000137 (PMC6380604; doi:10.1371/journal.pbio.3000137)

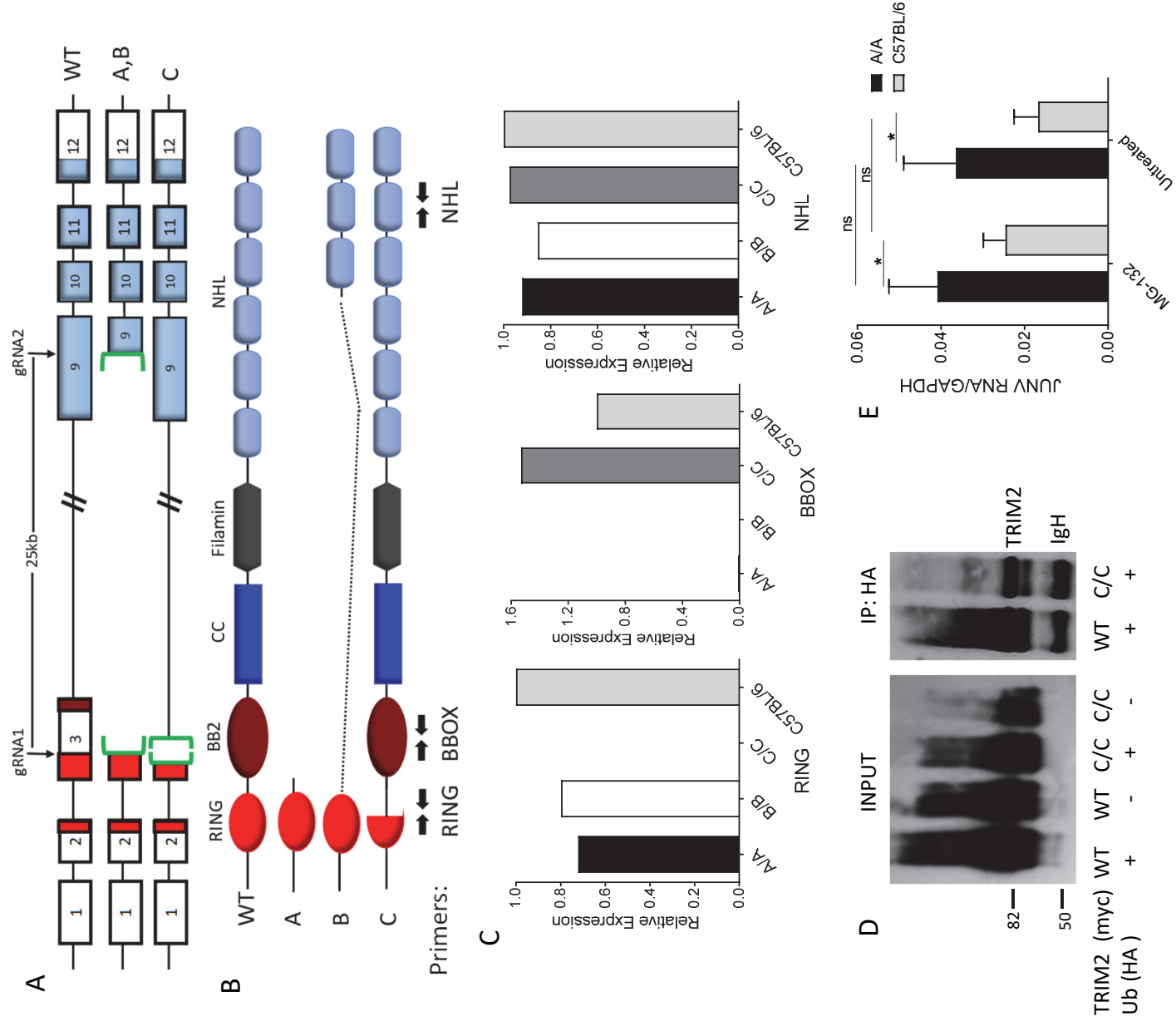

Figure S1

Supplement: S1 Fig — (A) Diagram showing the position of the guide RNAs used to generate the TRIM2 KO mice. Shown are the genomic deletions found in strains A, B, and C. (B) Diagram of the domains of TRIM2 present in the mutant mice. Shown are the primers used to analyze TRIM2 expression. (C) RT-qPCR analysis of RNA isolated from the brains of strains A, B, and C, using the indicated primers. (D) Ubiquitination assay performed with TRIM2 wild-type and strain C constructs. Immunoprecipitation was with anti-HA (Ub tag) and western blot with anti-myc (TRIM2 tag). (E) Primary macrophages isolated from strain A or C57BL/6 mice were treated with MG132 prior to and during infection. *P ≤ 0.03. One-way ANOVA was used to determine significance. HA, hemagglutinin; KO, knockout; RT-qPCR, real-time quantitative PCR; TRIM2, tripartite motif 2. (PDF) [file pbio.3000137.s001.pdf]

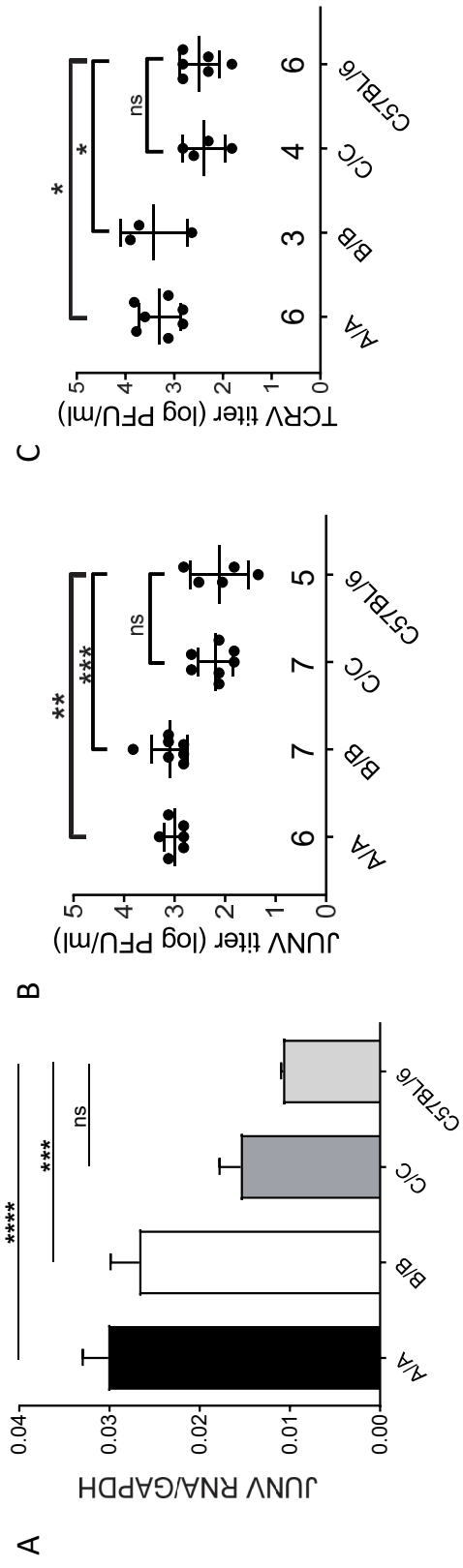

Figure S2

Supplement: S2 Fig — (A) Candid 1 infection of fibroblasts derived from strain A, B, and C mice. Shown are the averages ± SD of 3 different experiments. ***P ≤ 0.0005; ****P ≤ 0.0001. (B) Candid 1 titers in the brains of infected mice. Each symbol represents an individual mouse. Shown above the axis are the numbers of mice in each group. **P ≤ 0.003; ***P ≤ 0.0007. (C) Tacaribe virus titers in the spleens of infected mice. *P ≤ 0.02. One-way ANOVA was used to determine significance. (PDF) [file pbio.3000137.s002.pdf]

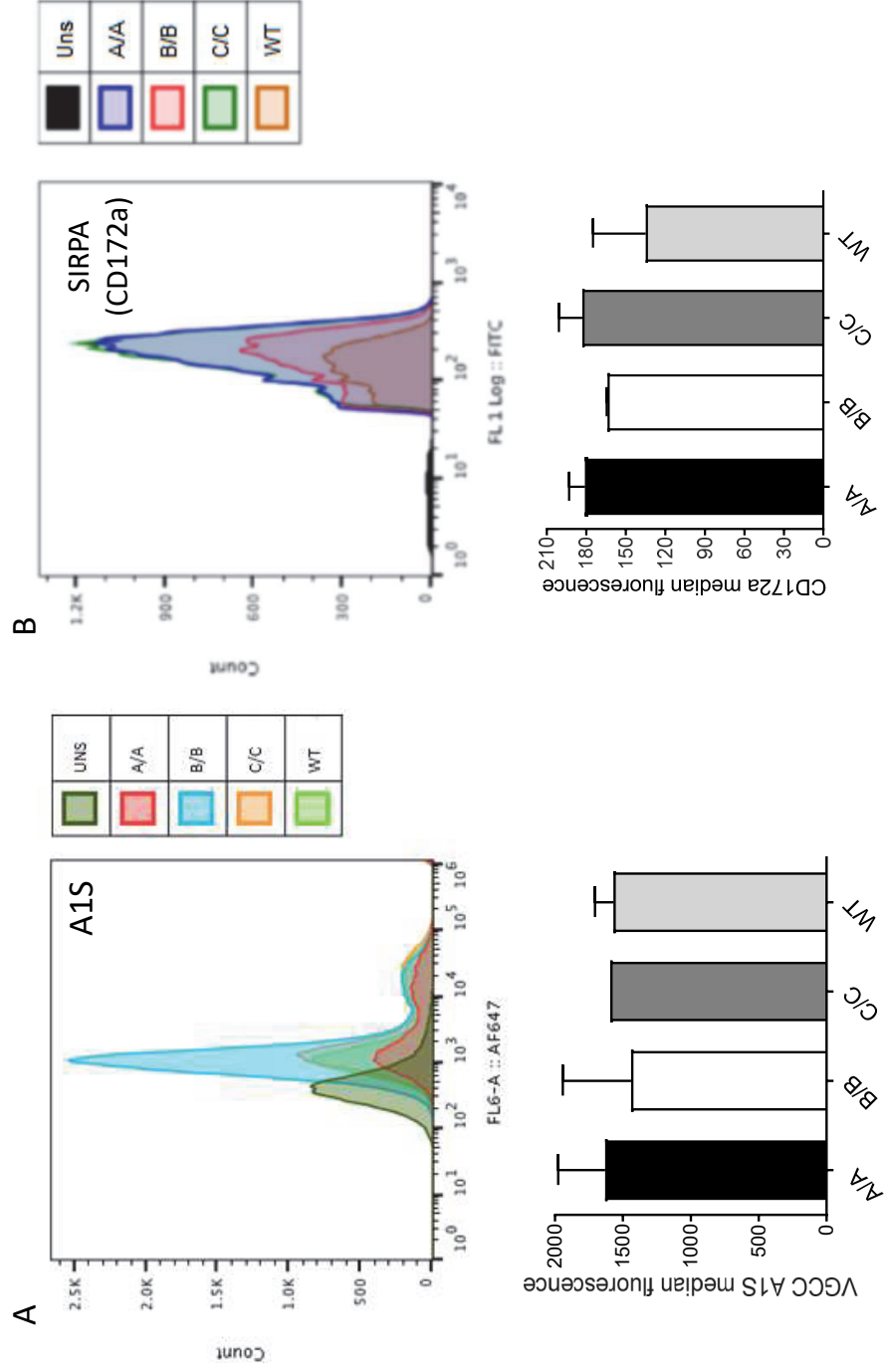

Figure S3

Supplement: S3 Fig — Primary macrophages from the indicated mice were stained with antibodies to the α1S subunit of the VGCC (anti-A1S) (A) and SIRPA (CD172a) (B). Shown below the histograms is the median fluorescence of BMDMs derived from 2 independent mice. BMDM, bone marrow–derived macrophage; SIRPA, signal regulatory protein α; VGCC, voltage-gated calcium channel. (PDF) [file pbio.3000137.s003.pdf]

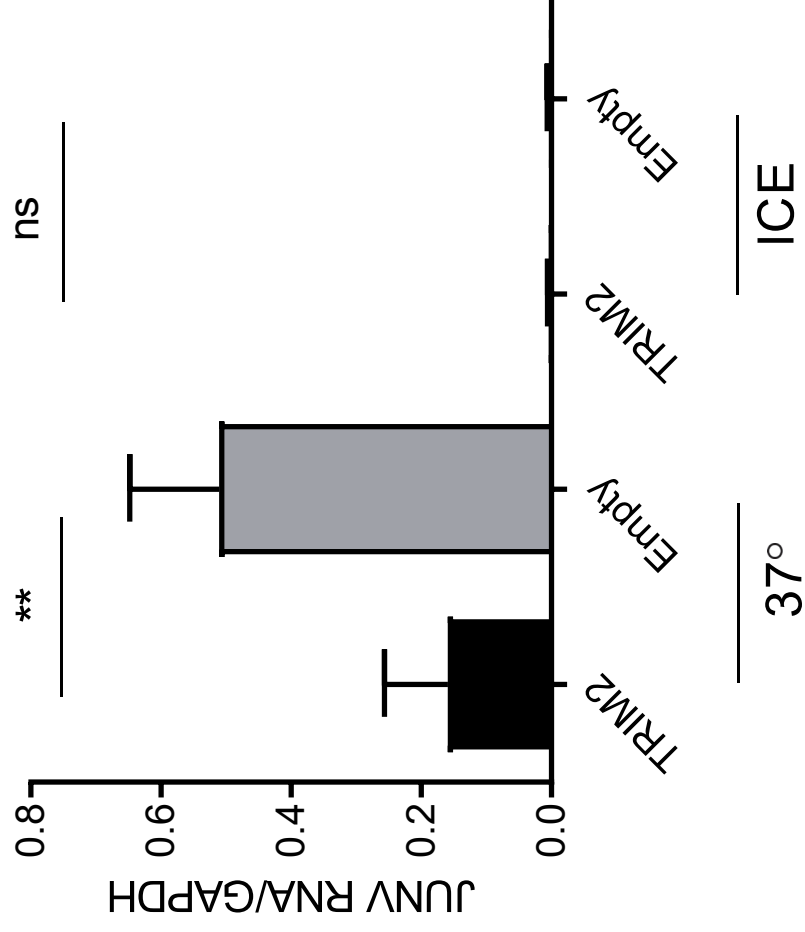

Figure S4

Supplement: S4 Fig — The same experiment as described in Fig 4B was performed, except that after virus binding on ice for 1 hr, the cells were incubated at 37°C or left on ice; the virus was stripped of all cells prior to RNA isolation. Shown are the averages ± SD of 3 different experiments. **P ≤ 0.004. One-way ANOVA was used to determine significance. TRIM2, tripartite motif 2. (PDF) [file pbio.3000137.s004.pdf]

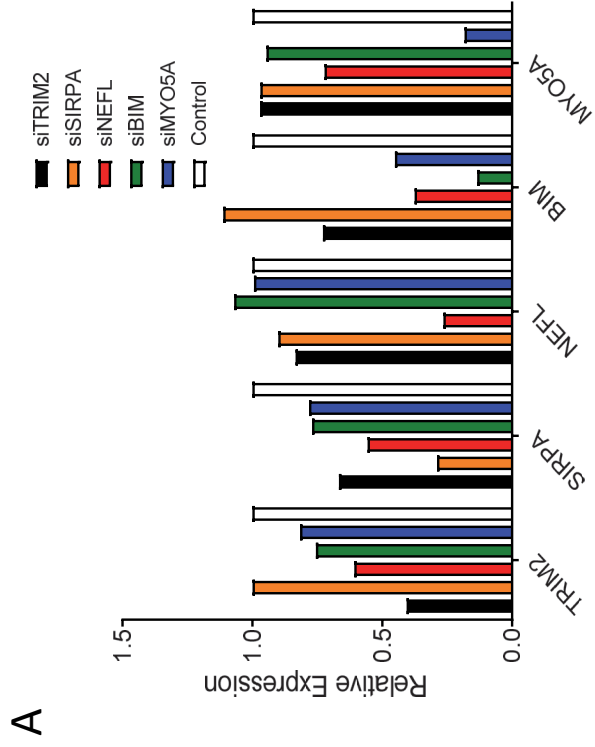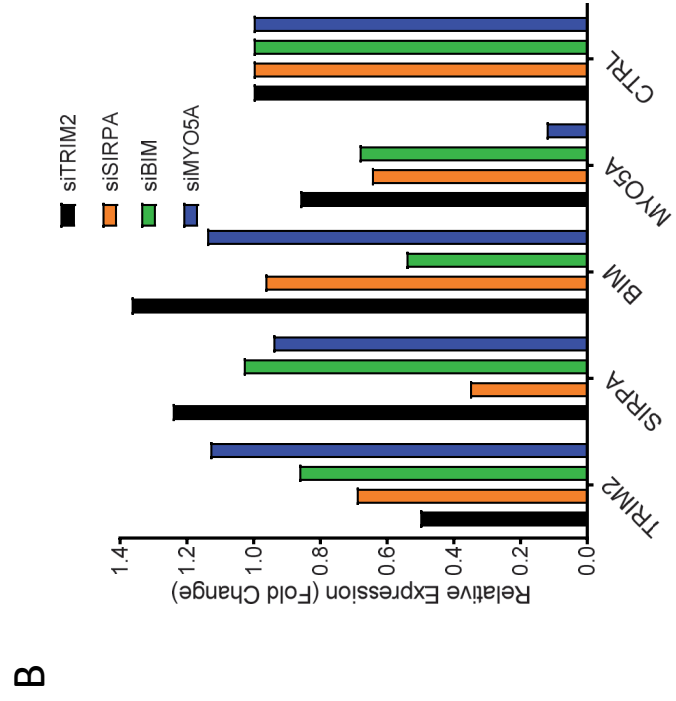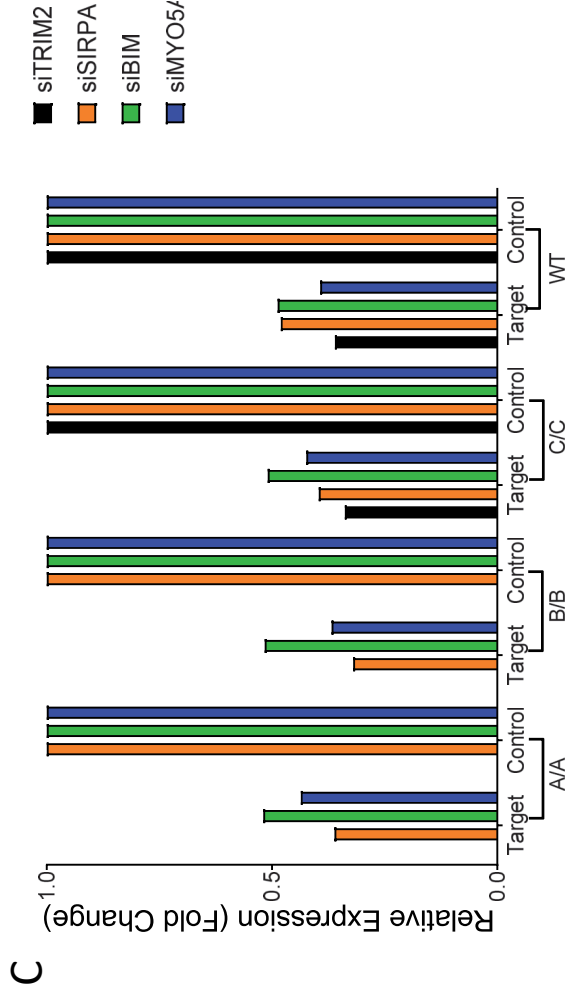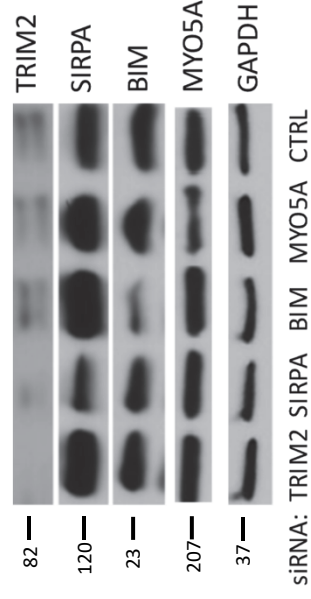

Figure S5

Supplement: S5 Fig — Panel A, Fig 6B; Panel B, Fig 6C (RNA, left; protein, right); Panel C, Fig 6D. (PDF) [file pbio.3000137.s005.pdf]

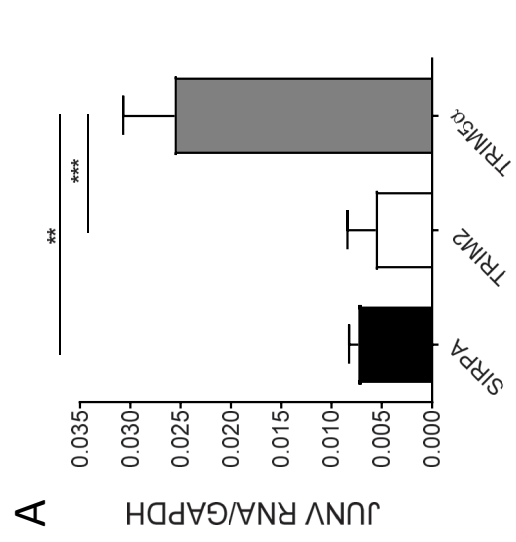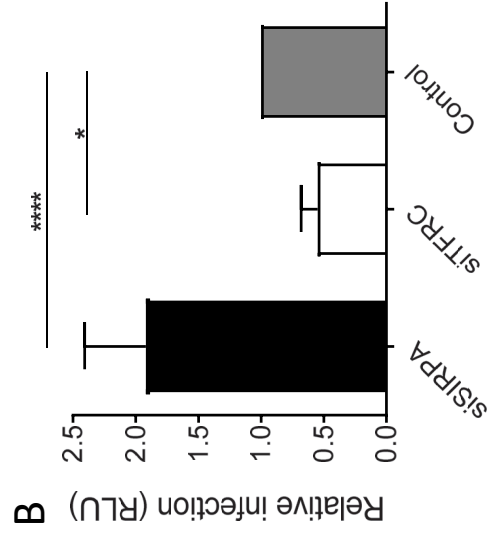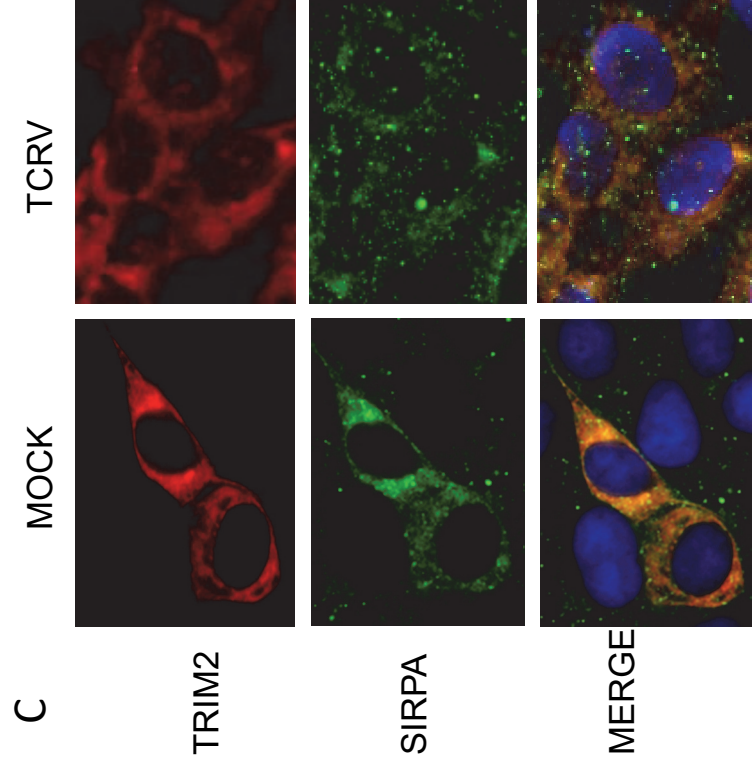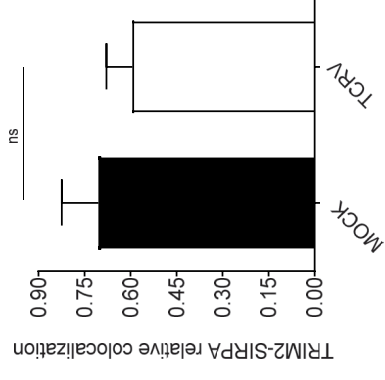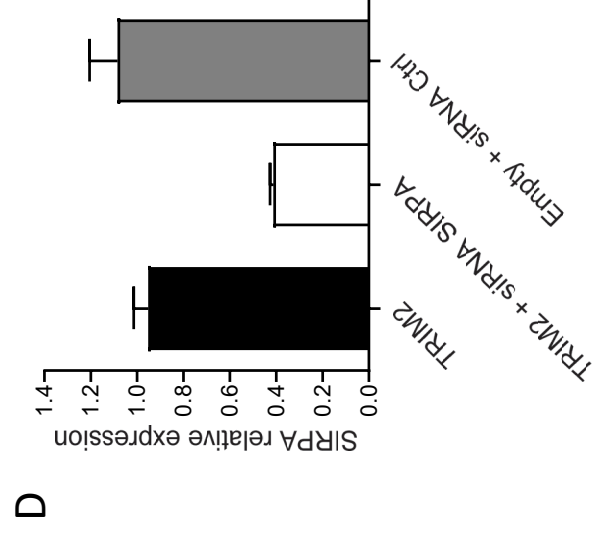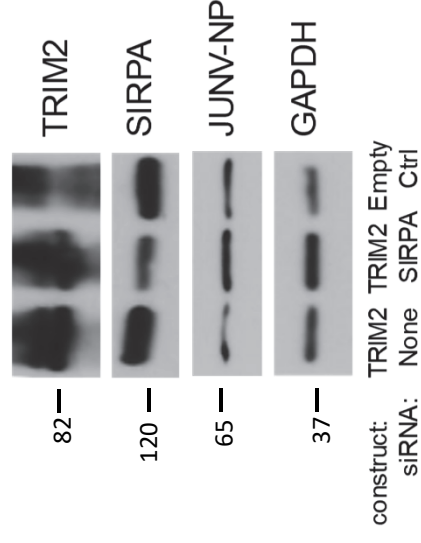

Figure S6

Supplement: S6 Fig — (A) U2OS cells were transfected with TRIM2, TRIM5α, or SIRPA expression vectors and 24 hr later infected with Candid 1 (MOI 0.1). RT-qPCR for the Junín NP was analyzed. Shown are the averages ± SDs of 3 independent experiments. One-way ANOVA was used to determine significance. **P ≤ 0.002; ***P ≤ 0.001. (B) U2OS cells were transfected with SIRPA, TfR1, or control siRNAs for 48 hr and infected with the Junín GP (Parodi)-pseudotyped MLV containing the luciferase gene. The data shown are the average and SDs of 8–10 replicates. One-way ANOVA was used to determine significance. ****P ≤ 0.0001; *P ≤ 0.01. (C) Immunostaining of U2OS cells cotransfected with TRIM2 and SIRPA expression vectors. Shown to the right is the quantification of TRIM2-SIRPA colocalization performed with 5 independent fields of each experiment and analyzed using the Coloc2 algorithm (ImageJ). (D) Knockdown control for Fig 7C (RNA, left; protein, right). GP, glycoprotein; MLV, murine leukemia virus; MOI, multiplicity of infection; NP, nucleoprotein; RT-qPCR, real-time quantitative PCR; siRNA, small interfering RNA; SIRPA, signal regulatory protein α; TfR1, transferrin receptor 1; TRIM, tripartite motif. (PDF) [file pbio.3000137.s006.pdf]

A

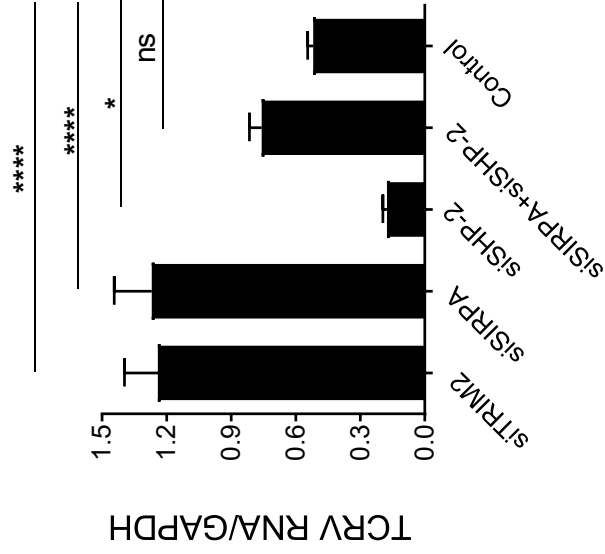

B

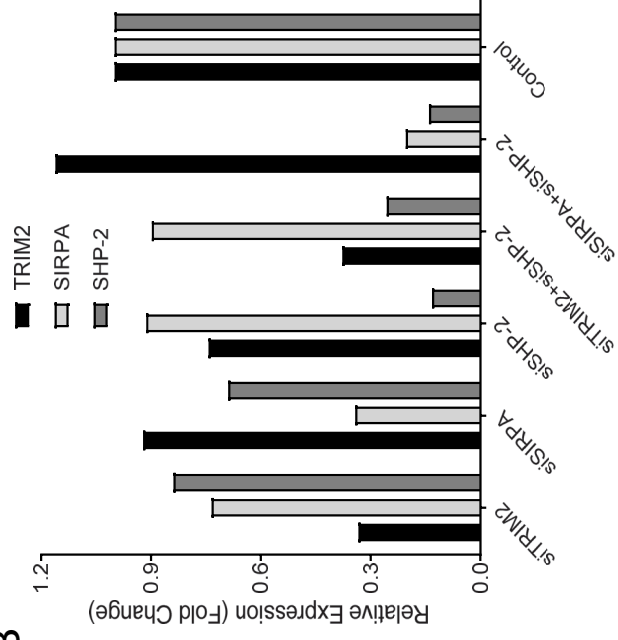

C

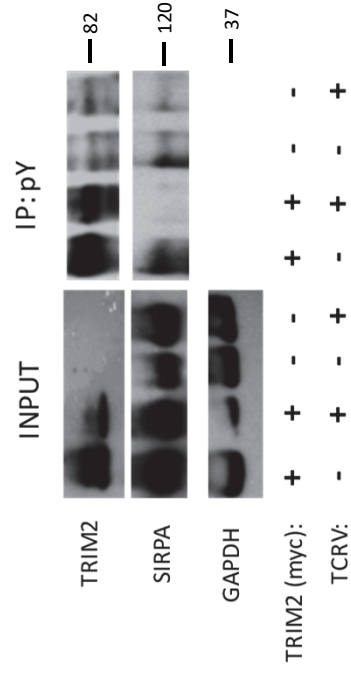

Figure S7

Supplement: S7 Fig — (A) U2OS cells were transfected with the indicated siRNAs and infected with Tacaribe virus, and RNA was isolated 24 hpi and analyzed for viral RNA. Values represent the average of 3 independent experiment ± SD. Statistical significance was calculated by one-way ANOVA. ****P ≤ 0.0001; *P ≤ 0.02. (B) Knockdown controls for Figs 8 and S7A. (C) U2OS cells were transfected with TRIM2 expression plasmid ± Tacaribe virus infection (MOI = 1). The extracts were immunoprecipitated with anti-phosphotyrosine antisera and analyzed by western blots with anti-myc (TRIM2) and a rabbit polyclonal anti-SIRPA. hpi, hours post infection; MOI, multiplicity of infection; TRIM2, tripartite motif 2. (PDF) [file pbio.3000137.s007.pdf]

A/A w/DEX-treated cells

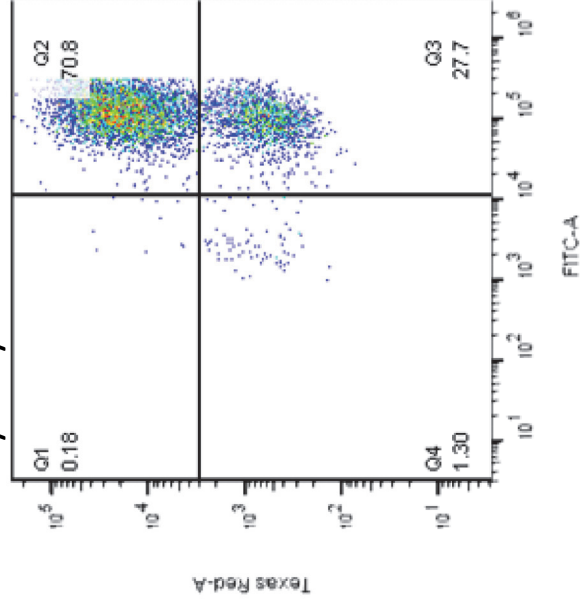

WT w/ DEX-treated cells

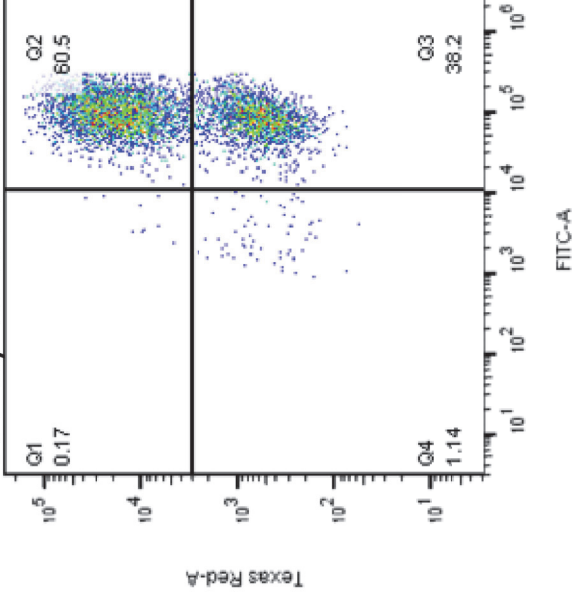

A/A w/live cells

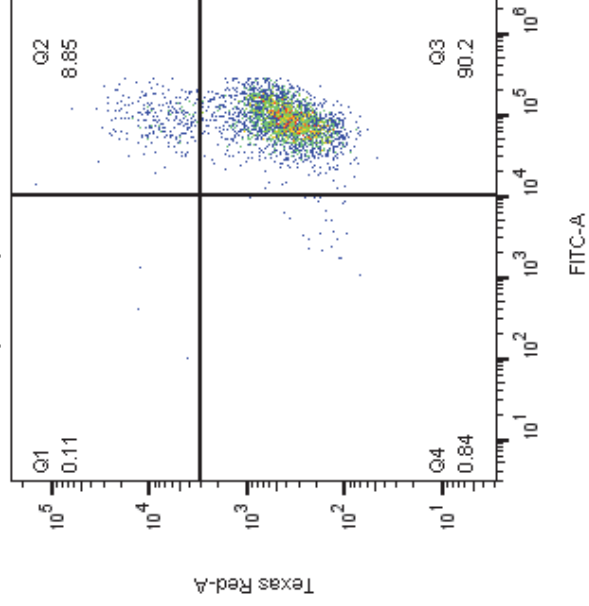

WT w/live cells

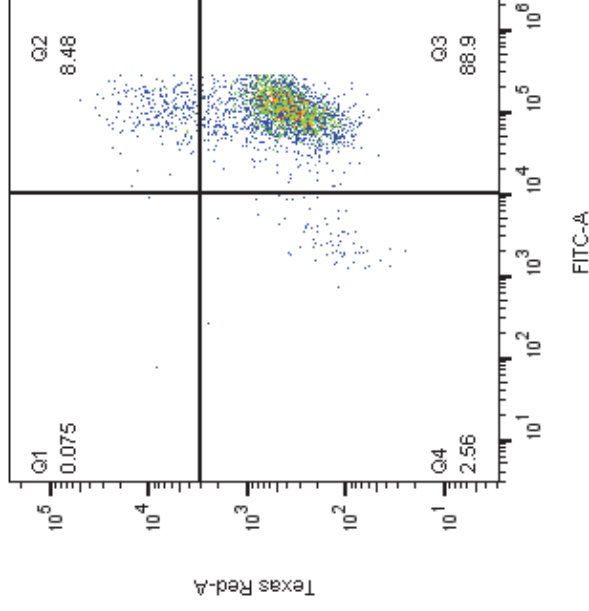

Figure S8

Supplement: S8 Fig — BMDM, bone marrow–derived macrophage; DEX, dexamethasone; FACS, fluorescence-activated cell sorting. (PDF) [file pbio.3000137.s008.pdf]
