# Supplementary material for: TRIM2, a novel member of the antiviral family, limits New World arenavirus entry
Source: PLoS Biol. 2019 Feb 6;17(2):e3000137. doi: 10.1371/journal.pbio.3000137 (PMC6380604; doi:10.1371/journal.pbio.3000137)
Supplement: S1 Table — RT-qPCR, real-time quantitative PCR. (DOCX) [file pbio.3000137.s009.docx]

| Sequence Name | Sequence |
| --- | --- |
| JUNV-SF | GGGGCAGTTCATTAGCTTCATGC |
| JUNV-SR | CAAAGGTAGGTCATGTGGATTGTTGG |
| LCMV-SF | AGA ATC CAG GTG GTT ATT GCC |
| LCMV-SR | GTT GTA GTC AAT TAG TCG CAG C |
| VSV-NP-F | TGA ATG TGC CTC GTT CAG ATA |
| VSV-NP-R | CCA AAG TCG ATC AAA TAA GGC |
| BIM-F | ATG TCT GAC TCT GAC TCT CGG |
| BIM-R | CTC CGT GAT TGC CTT CAG GAT |
| SIRPA-F | TTC CAG TGC CTT CCA GCC CT |
| SIRPA-R | GGT GAT GTT ACC GAT GCG GAT G |
| MYO5A-F | GCT CCA TAA TCT CAG AGT CCG |
| MYO5A-R | ATG TTC TGA CCA CTG TAT GC |
| NEFL-F | TGG TTC AAG AGC CGC TTC ACC |
| NEFL-R | GCC ATC TTC ACG TTG AGG AGG |
| TCRV-GPF | TCG GTC ACA GAT GGG ACC AGG |
| TCRV-GPR | CAG GGT TCT TCA CGT CCT CTG |
| mTRIM2-F2 | TGT CTG CAC ACT TTC TGC GAG AG |
| mTRIM2-R2 | GTT TGG GCA CGA AAG AGG CTT TC |
| TRIM2-RINGF | CAG CAT CCC AAG TCC CGT GGT |
| TRIM2-RINGR | TCT CGC AGA AAG TGT GCA GAC AG |
| TRIM2-NHL5F | ATG TTG AAG TTT GGC TCC AAT |
| TRIM2-NHL5R | CAG CAG ATG TGT TGA TAT AGG |
| mSIRPA-For | CAT CCA GCC AGC CAA TCC TGT |
| mSIRPA-Rev | TCC AGT TCG CCC TCT GGT TCT |
| mBIM-For | AGC AAC CTT CTG ATG TAA GTT CTG |
| mBIM-Rev | GGT TCT GTC TGT AGG GAG GTA |
| SHP-2 For | TGG TCC AGT ATT ACA TGG AAC |
| SHP-2 Rev | TCA CTT TAG ACT TGC CGT CAT |
| GAPDH F | CCCCTTCATTGAC CTCAACTACA |
| GAPDH r | CGCTCCTGGAGGATGGTGAT |

S1 Table. Primer pairs used for reverse-transcribed RT-qPCR.
